# *Caenorhabditis elegans* orthologs of human genes differentially expressed with age are enriched for determinants of longevity

**DOI:** 10.1111/acel.12595

**Published:** 2017-04-12

**Authors:** George L. Sutphin, Grant Backer, Susan Sheehan, Shannon Bean, Caroline Corban, Teresa Liu, Marjolein J. Peters, Joyce B. J. van Meurs, Joanne M. Murabito, Andrew D. Johnson, Ron Korstanje, the Cohorts for Heart and Aging Research in Genomic Epidemiology (CHARGE) Consortium Gene Expression Working Group

**Affiliations:** ^1^ The Jackson Laboratory 600 Main Street Bar Harbor ME 04609 USA; ^2^ Department of Internal Medicine Erasmus Medical Center Postbus 2040 3000 CA Rotterdam The Netherlands; ^3^ Section of General Internal Medicine Boston University School of Medicine 801 Massachusetts Ave, Crosstown Center Boston MA 02118 USA; ^4^ The National Heart, Lung, and Blood Institute's Framingham Heart Study 73 Mt. Wayte Ave, Suite 2 Framingham MA 01702‐5827 USA; ^5^ Population Sciences Branch National Heart, Lung, and Blood Institute Building 31, Room 5A52, 31 Center Drive MSC 2486 Bethesda MD 20892 USA

**Keywords:** aging, *Caenorhabditis elegans*, comparative genetics, *Homo sapiens*, lifespan, transcriptomics

## Abstract

We report a systematic RNAi longevity screen of 82 *Caenorhabditis elegans* genes selected based on orthology to human genes differentially expressed with age. We find substantial enrichment in genes for which knockdown increased lifespan. This enrichment is markedly higher than published genomewide longevity screens in *C. elegans* and similar to screens that preselected candidates based on longevity‐correlated metrics (e.g., stress resistance). Of the 50 genes that affected lifespan, 46 were previously unreported. The five genes with the greatest impact on lifespan (>20% extension) encode the enzyme kynureninase (*kynu‐1*), a neuronal leucine‐rich repeat protein (*iglr‐1*), a tetraspanin (*tsp‐3*), a regulator of calcineurin (*rcan‐1*), and a voltage‐gated calcium channel subunit (*unc‐36*). Knockdown of each gene extended healthspan without impairing reproduction. *kynu‐1*(*RNAi*) alone delayed pathology in *C. elegans* models of Alzheimer's disease and Huntington's disease. Each gene displayed a distinct pattern of interaction with known aging pathways. In the context of published work, *kynu‐1*,* tsp‐3*, and *rcan‐1* are of particular interest for immediate follow‐up. *kynu‐1* is an understudied member of the kynurenine metabolic pathway with a mechanistically distinct impact on lifespan. Our data suggest that *tsp‐3* is a novel modulator of hypoxic signaling and *rcan‐1* is a context‐specific calcineurin regulator. Our results validate *C. elegans* as a comparative tool for prioritizing human candidate aging genes, confirm age‐associated gene expression data as valuable source of novel longevity determinants, and prioritize select genes for mechanistic follow‐up.

## Introduction

Understanding which molecular processes contribute to aging is critical to developing interventions capable of extending healthy human lifespan and delaying onset of age‐associated diseases. A key step in this process is building a comprehensive model encompassing the range of genetic and environmental factors that influence lifespan and describing the complex interaction between these factors in an aging organism. Directly screening interventions for lifespan phenotypes in mammals is limited by long lifespans. Despite evolutionary distance and orders‐of‐magnitude differences in lifespan, processes that contribute to aging are sufficiently conserved that mechanistic knowledge gleaned from short‐lived invertebrates can be beneficially applied to mammalian systems. Genetic screens in the nematode, *Caenorhabditis elegans*, have identified hundreds of genes capable of influencing lifespan (Yanos *et al*., 2012; Sutphin & Korstanje, 2016). Pharmacological agents identified as prolongevity using invertebrate models – rapamycin, metformin, resveratrol – are now in clinical trials for treatment of age‐associated disease in humans (Kennedy & Pennypacker, 2015).

An approach that is tractable in humans is to characterize systemic changes that occur during normal aging. This approach identifies traits that change with age or during age‐associated disease and employs targeted studies to determine which play a causative role in aging. Early applications focused on easily measurable physiological traits, such as body weight or circulating molecules, but has now expanded into the ‘‐omics’ realm to provide systems‐level insight into molecular changes that occur with age. As part of the Cohorts for Heart and Aging Research in Genomic Epidemiology (CHARGE) Consortium, we published a large meta‐analysis of gene expression in human peripheral blood from 14,983 individuals representing ages across the adult lifespan (Peters *et al*., 2015). This study identified 1,497 genes with significantly different expression at different ages. Gene sets with a defined age‐associated expression pattern provide information about molecular processes with altered activity during aging and provide a valuable diagnostic tool for determining individual biological rate of aging and predicting risk of age‐associated disease, as demonstrated in follow‐up analyses (Peters *et al*., 2015). On a gene‐by‐gene basis, differential expression alone is insufficient to distinguish between genes that play a causative role in aging and genes that merely respond to the altered physiological environment in an aging organism.

In this study, we selected the human genes with the most significant differential expression with age from the CHARGE meta‐analysis and used RNAi to screen *C. elegans* orthologs for lifespan phenotypes. This selection criterion ensured that every gene identified in the lifespan screen was already of interest in the context of human aging. The short lifespan of *C. elegans* allowed genes capable of directly influencing lifespan to be rapidly identified and characterized. The resulting *C. elegans* candidate list was substantially enriched in genes for which knockdown extends lifespan. We selected the five genes with the greatest impact on lifespan for additional characterization and found that each gene produced a distinct pattern of influence on healthspan, reproduction, neurodegenerative pathology, and interaction with established aging pathways.

## Results

### Gene selection

We began by selecting the 125 genes with the most significant differential expression with age in human peripheral blood from the 1,497 identified in the CHARGE study (Peters *et al*., 2015). Each gene was replicated in independent samples with significant meta‐analysis *P*‐values ranging from 1.98 × 10^−59^ to 1.62 × 10^−577^ (Table S1, Supporting information). Our initial goal was to determine which genes were capable of directly influencing lifespan in *C. elegans*. We identified *C. elegans* orthologs for each human gene using the WORMHOLE ortholog prediction tool (Sutphin *et al*., 2016), yielding 88 *C. elegans* orthologs corresponding to 61 of the 125 human candidate genes (Tables 1 and S2, Supporting information). We obtained feeding RNAi bacterial strains with the correct target sequence for 82 of the 88 *C. elegans* genes, forming the ‘CHARGE gene set’. The CHARGE gene set includes 37 high‐confidence orthologs (HCOs) and 45 related proteins (RPs) (Table S2, Supporting information; see Experimental procedures). As a control, we selected 59 *C. elegans* genes (25 HCOs and 34 RPs) based on orthology to randomly selected human genes – the ‘Random gene set’ (Tables 1 and S3, Supporting information).

**Table 1:** The CHARGE gene set is enriched in genes for which knockdown extends lifespan relative to the random gene set at 25 °C

Orthologs included	All				HCO only			
Temperature	15 °C		25 °C		15 °C		25 °C	
Gene set	CHARGE	Random	CHARGE	Random	CHARGE	Random	CHARGE	Random
Human genes (all)	125	281	125	281	125	281	125	281
Human genes with worm ortholog(s)	61	63	61	63	45	39	45	39
Worm orthologs identified	88	160	88	160	39	37	39	37
Worm orthologs tested^a^	82	59	82	59	37	25	37	25
# long‐lived RNAi	5	7	36	4	3	2	15	1
# not long‐lived RNAi	77	52	46	55	34	23	22	24
% long‐lived	6.1%	11.7%	43.9%	6.7%	6.7%	9.1%	46.7%	9.1%
Odds Ratio^b^	0.49		10.79		1.01		15.78
*P*‐value (Fisher's Exact Test)^b^	0.360		4.70E‐07		1.000		1.06E‐03

HCO, high‐confidence ortholog.

a Sequence‐verified RNAi available.

b Enrichment for long‐lived RNAi in CHARGE vs. Random gene sets.

### The charge gene set is enriched for determinants of lifespan at 25 °C

The efficacy of many longevity interventions is strongly dependent on environmental temperature (Lakowski & Hekimi, 1996; Gems *et al*., 1998; Lee & Kenyon, 2009; Leiser *et al*., 2011; Sutphin *et al*., 2012; Horikawa *et al*., 2015; Zhang *et al*., 2015). To capture genes that influence longevity across the temperature spectrum, we employed a two‐temperature screening strategy, measuring lifespan of wild‐type (N2) worms subjected to RNAi targeting each gene at 15 and 25 °C. We initially measured lifespan for ~105 worms per gene at each temperature and validated each RNAi that significantly extended lifespan relative to experiment‐matched empty vector (EV) RNAi through at least two additional rounds of lifespan measurement.

**Figure 1:**
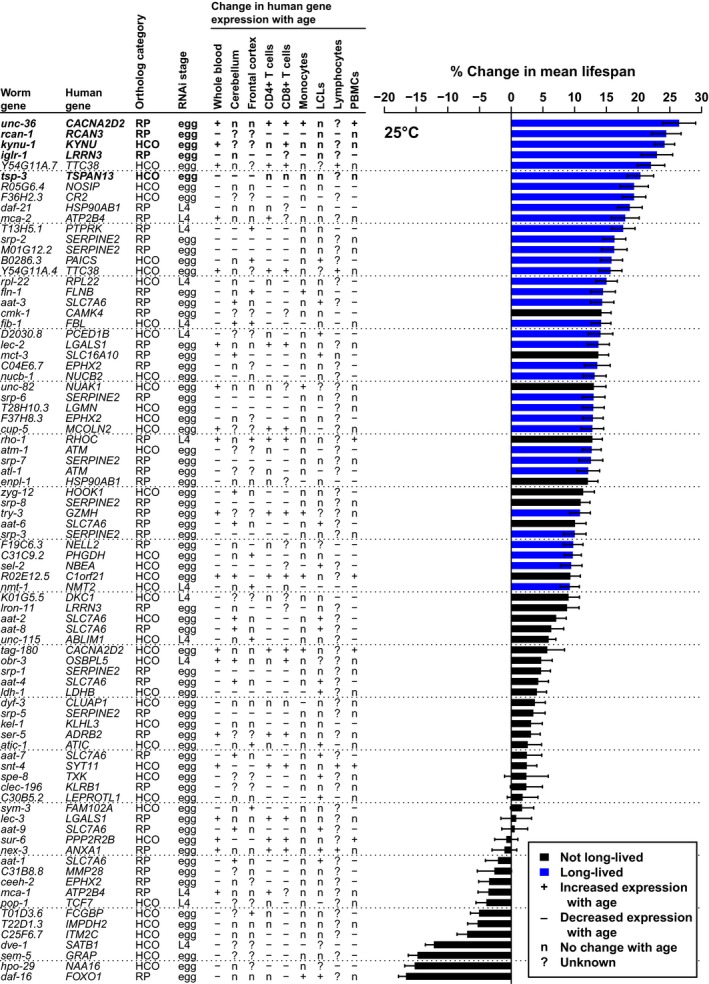
RNAi knockdown of 36 of 82 genes in CHARGE gene set increases lifespan at 25 °C. Bars indicate percent change in mean lifespan for RNAi targeting CHARGE genes relative to experiment‐matched *EV*(*RNAi*) when experiments were pooled. Error bars are standard error. Blue bars indicate RNAi clones that increased lifespan by our significance criteria. For each *Caenorhabditis elegans* gene, the table to the left indicates the corresponding human ortholog, the ortholog confidence category (high‐confidence ortholog, HCO; related protein, RP), the life stage at which RNAi was started, and the direction of change in expression of each human gene with age in specified tissues [lymphoblastoid cell line, LCL; peripheral blood mononuclear cell, PBMC; assembled from Peters *et al*. (2015)].

The CHARGE gene set was significantly enriched for RNAi capable of increasing lifespan relative to the Random gene set at 25 °C (*P* < 4.70 × 10^−7^), but not at 15 °C (*P* = 0.360) (Table 1). Of the 82 CHARGE genes, RNAi knockdown of 36 (43.9%) increased lifespan at 25 °C (Figs 1 and S2; Table S4, Supporting information), while RNAi knockdown of only five (6.1%) increased lifespan at 15 °C (Figs S1 and S2; Table S4, Supporting information). In contrast, RNAi knockdown of only four (6.7%) or seven (11.7%) of the 59 Random genes increased lifespan at 25 or 15 °C, respectively (Fig. S3, Supporting information; Tables 1 and S5, Supporting information). Of the 50 genes identified in either the CHARGE or Random gene sets, only 4 – *atl‐1* (Suetomi *et al*., 2013), *atm‐1* (Ventura *et al*., 2009), *daf‐21* (Horikawa *et al*., 2015), and *tag‐322* (Bell *et al*., 2009) – were previously reported to affect lifespan in *C. elegans*, while the remaining 46 are novel.

**Figure 2:**
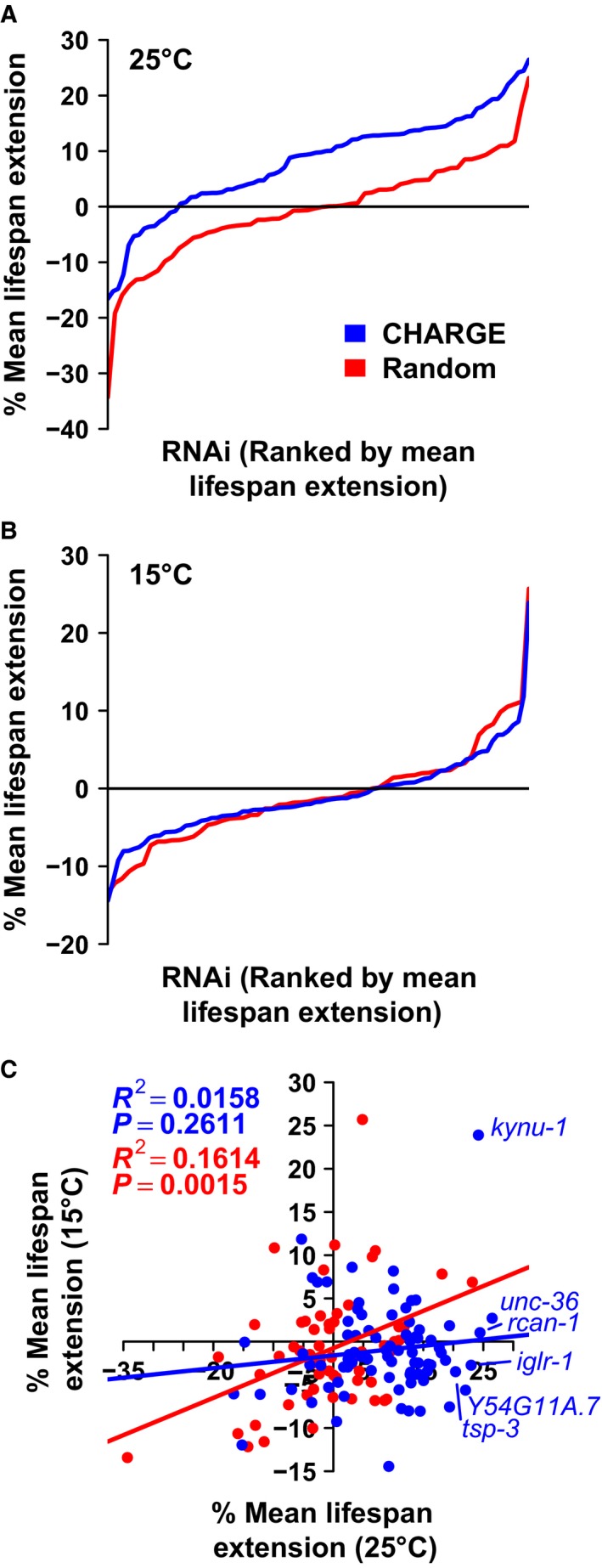
CHARGE gene set is enriched for longevity determinants at 25 °C. Worms fed RNAi targeting CHARGE genes lived significantly longer than worms fed RNAi targeting Random genes at 25 °C (*P* < 0.0001) (A), but not at 15 °C (*P* = 0.927, linear mixed‐effects model) (B). Lines represent mean percent lifespan extension for candidate RNAi relative to experiment‐matched *EV*(*RNAi*). RNAi are rank‐ordered by mean lifespan extension (left to right). (C) Lifespan extension at 15 and 25 °C is significantly correlated for Random, but not CHARGE genes (*R* = Pearson correlation coefficient).

To examine the effect of gene set on lifespan, we built a linear mixed model with target gene and experiment as nested random effects. Worms subjected to RNAi targeting CHARGE genes were significantly longer‐lived than worms subjected to RNAi targeting Random genes at 25 °C (β = 0.045, *P* < 0.0001), but not at 15 °C (β = −0.001, *P* = 0.927) (Fig. 2A,B; Table S6, Supporting information). The degree of lifespan extension at 15 °C vs. 25 °C correlated significantly for Random genes (*R* ^2^ = 0.161, *P* < 0.005), but not for CHARGE genes (*R* ^2^ = 0.016, *P* = 0.261) (Fig. 2C). Combined, these data show that our candidate selection strategy specifically enriched for genes that influence long‐term survival of worms at high temperature.

### Knocking down genes with the greatest lifespan extension increases healthspan without impairing reproduction

**Figure 3:**
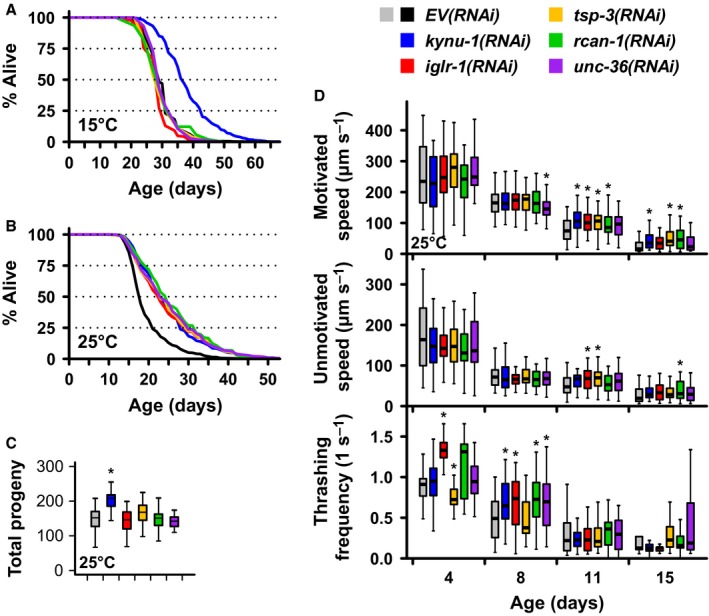
RNAi knockdown of selected genes differentially affects lifespan, reproduction, and healthspan. (A) RNAi knockdown of *kynu‐1*,* iglr‐1*,* tsp‐3*,* rcan‐1*, or *unc‐36* extends lifespan at 25 °C. *kynu‐1*(*RNAi*) alone (B) extends lifespan at 15 °C and (C) increases total brood size at 25 °C. (D) RNAi knockdown of *kynu‐1*,* iglr‐1*,* tsp‐3*, or *rcan‐1*, but not *unc‐36* delays decline in motivated speed with age (top), while unmotivated speed is largely unaffected (middle). RNAi knockdown of *kynu‐1*,* iglr‐1*,* rcan‐1*, or *unc‐36*, but not *tsp‐3*, increases thrashing rate in liquid early in life (bottom). *P < 0.05 vs. age‐matched *EV*(*RNAi*). For box and whisker plots, center line indicates median, boxes indicate 25th and 75th percentiles, and whiskers indicate 5th and 95th percentiles.

To extend the most significant outcomes from our screen, we further characterized the genes with the greatest impact on lifespan. Knockdown of five genes increased lifespan by >20% at 25 °C (Figs 1 and 3A; Table S4, Supporting information) while maintaining or extending lifespan at 15 °C (Figs 3B and S1; Table S5, Supporting information): *kynu‐1* (previously *flu‐*2) encodes the kynurenine pathway enzyme kynureninase (KYNU), *iglr‐1* encodes a neuronal leucine‐rich repeat (NLRR) protein, *tsp‐3* encodes a tetraspanin, *rcan‐1* encodes a regulator of calcineurin, and *unc‐36* encodes a voltage‐gated calcium channel subunit. We selected these genes for further analysis.

To validate the lifespan extension observed in the RNAi screen, we obtained null mutants for each gene. Each mutation extended lifespan to a similar degree as the corresponding RNAi (Fig. S4A–E; Table S7, Supporting information), with the exception of *unc‐36*. *unc‐36* deletion impaired development, resulting in small adult body size and short lifespan (Fig. S4F; Table S7, Supporting information). Maintaining wild‐type worms on *unc‐36*(*RNAi*) for four generations prior to measuring lifespan partially replicated the *unc‐36* mutant lifespan (Fig. S4G; Table S7, Supporting information), suggesting that functional UNC‐36 is required maternally or during early life (before larval stage 1) for normal development. *kynu‐1* deletion resulted in a high‐penetrant temperature‐dependent developmental arrest. At 15 °C, *kynu‐1* null worms that reached adulthood were small and long‐lived relative to wild‐type, but not as long‐lived as wild‐type worms subjected to *kynu‐1*(*RNAi*) from egg (Fig. S4B; Table S7, Supporting information). Maintaining wild‐type worms on *kynu‐1*(*RNAi*) for four generations replicated the lifespan phenotype of the mutant strain (Fig. S4H; Table S7, Supporting information) with a less penetrant developmental arrest, suggesting that KYNU‐1 is required for normal development at 15 °C. This is consistent with past studies that find enrichment for lifespan extension in response to RNAi knockdown of genes required for development (Chen *et al*., 2007; Curran & Ruvkun, 2007).

We next measured phenotypes commonly associated with increased lifespan. Antagonistic pleiotropy predicts that genes inferring increased late‐life mortality may be selected if they provide early‐life benefits. Accordingly, many *C. elegans* longevity mutants have defects in reproduction. Surprisingly, none of the candidate RNAi reduced the number of progeny produced, and *kynu‐1*(*RNAi*) even increased total brood size at 25 °C (Figs 3C and S4A; Table S8, Supporting information). This result does not discount antagonistic pleiotropy, as reproductive ability contributes to, but is not identical with, overall fitness.

Extending lifespan without delaying functional decline is undesirable. We measured the impact of each candidate RNAi on three healthspan phenotypes: (i) motivated movement on solid media (average speed following a plate tap), (ii) unmotivated movement on solid media (average speed without stimulation), and (iii) thrashing frequency in liquid. Each candidate RNAi significantly delayed the decline in motivated speed, thrashing frequency, or both, while unmotivated speed was largely unaffected (Fig. 3D; Table S9, Supporting information). Knocking down each gene thus allows worms to retain the ability to move later in life without affecting behavior.

Of the five candidate human/worm gene pairs, *KYNU*/*kynu‐1, LRRN3*/*iglr‐1*,* RCAN3/rcan‐1*, and *CACNA2D2*/*unc‐36* have been implicated in neurological function and/or neurodegeneration. In *C. elegans*, neurodegenerative disease is modeled by expressing toxic aggregate‐prone peptides in body wall muscle, resulting in age‐dependent paralysis and aggregate formation. We measured age‐dependent paralysis in strains expressing amyloid‐beta (Aβ; modeling Alzheimer's disease) or a 35‐unit polyglutamine repeat (Q35; modeling Huntington's disease) at 25 °C. None of the candidate RNAi delayed paralysis in either model, except *kynu‐1*(*RNAi*), which slightly delayed paralysis in Aβ worms (Fig. S6A; Table S10, Supporting information).

### Each candidate interacts with distinct combinations of established aging pathways

We next applied RNAi targeting our top candidate genes to mutant worms representing five established aging pathways to look for genetic interaction. For insulin signaling and the hypoxic response, we used mutant worms lacking the mediating transcription factors DAF‐16 and HIF‐1, respectively; for dietary restriction (DR), we used *eat‐2* mutants, a genetic model of DR with reduced food intake; for mTOR signaling, we used worms lacking RSKS‐1, the ribosomal protein S6 kinase (S6K); and for sirtuins, we used worms lacking the *C. elegans* sirtuin *sir‐2.1*. Specifically, we asked whether each candidate RNAi was capable of extending lifespan in each aging mutant.

**Figure 4:**
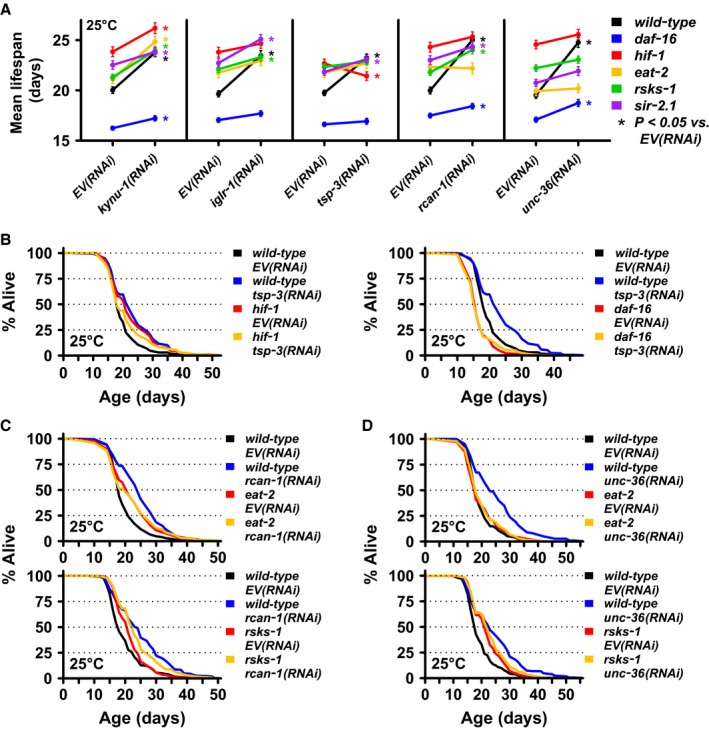
Each candidate aging gene interacts with known aging pathways in a distinct manner. (A) Summary of lifespan interaction between *kynu‐1*,* iglr‐1*,* tsp‐3*,* rcan‐1*, or *unc‐36* and aging pathway mutants: insulin signaling (*daf‐16*), hypoxic response (*hif‐1*), DR (*eat‐2*), mTOR signaling (*rsks‐1*), and sirtuins (*sir‐2.1*). Each point represents the mean lifespan for an RNAi (*x*‐axis) applied to a specific strain (line color) for pooled data. Error bars are standard error. *P < 0.05 vs. *EV*(*RNAi*) (Wilcoxon rank sum test with Bonferroni multiple test correction). (B) Survival curves for *tsp‐3*(*RNAi*) in *hif‐1* (left) or *daf‐16* (right) mutants. (C) Survival curves for *rcan‐1*(*RNAi*) in *eat‐2* (top) or *rsks‐1* (bottom) mutants. (D) Survival curves for *unc‐36*(*RNAi*) in *eat‐2* (top) or *rsks‐1* (bottom) mutants.

Each candidate gene displayed a distinct pattern of interaction with the aging pathway genes (Figs 4A and S7; Table S11, Supporting information). Lifespan extension from *tsp‐3*(*RNAi*) was completely prevented in *daf‐16* mutants and slightly reversed in *hif‐1* mutants, suggesting that *tsp‐3* may influence lifespan, at least in part, by activating the hypoxic response (Fig. 4B). Both *rcan‐1*(*RNAi*) (Fig. 4C) and *unc‐36*(*RNAi*) (Fig. 4D) failed to extend lifespan of *eat‐2* mutant worms, suggesting that both genes may extend lifespan in a manner similar to DR. Supporting this model – mTOR signaling is thought to mediate the beneficial effects of DR on lifespan – knockout of *rsks‐1* partially repressed lifespan extension from *rcan‐1*(*RNAi*) and prevented lifespan extension from *unc‐36*(*RNAi*) (Fig. 4C,D).

*iglr‐1*(*RNAi*) failed to extend lifespan in any mutant background except *sir‐2.1*. In most cases, the lifespan of the aging pathway mutant fed *iglr‐1*(*RNAi*) was similar to or longer than wild‐type worms fed *iglr‐1*(*RNAi*) (Fig. 4A). This type of nonadditive interaction between lifespan‐extending interventions is difficult to interpret. The exception was the *daf‐16* mutant, which had a short lifespan that was not extended by *iglr‐1*(*RNAi*), suggesting that *iglr‐1* knockdown requires DAF‐16 to extend lifespan.

Among the candidates examined, knockdown of *kynu‐1* alone significantly extended lifespan in all aging mutants (Fig. 4A). van der Goot *et al*. (2012) reported that RNAi knockdown of *tdo‐2*, encoding the upstream kynurenine pathway enzyme tryptophan (TRP) 2,3‐dioxygenase (TDO), increased lifespan and delayed neurodegenerative pathology in *C. elegans*. In contrast to *kynu‐1*(*RNAi*), lifespan extension from *tdo‐2*(*RNAi*) was largely dependent on *daf‐16* and independent of *eat‐2*. They also report a protracted reproductive period for *tdo‐2*(*RNAi*), in contrast to the slightly increased brood size that we observed for *kynu‐1*(*RNAi*) (Figs 3C and S5A, Supporting information). To confirm that the RNAi was producing the expected metabolic perturbations (Fig. S8A, Supporting information) we measured kynurenine pathway metabolites and observed the expected increase in TRP in worms subjected to *tdo‐2*(*RNAi*), and in both kynurenine (KYN) and 3‐hydroxykynurenine (3HK) in worms subjected to *kynu‐1*(*RNAi*) (Fig. S8B, Supporting information). Our experiments with *kynu‐1*(*RNAi*) were conducted at 25 °C in a wild‐type background, while van der Goot *et al*. (2012) examined *tdo‐2*(*RNAi*) at 20 °C in a transgenic α‐synuclein background. To determine whether experimental context was responsible for the observed differences, we examined each phenotype using identical environmental conditions (15 or 25 °C) and genetic background (wild type).

**Figure 5:**
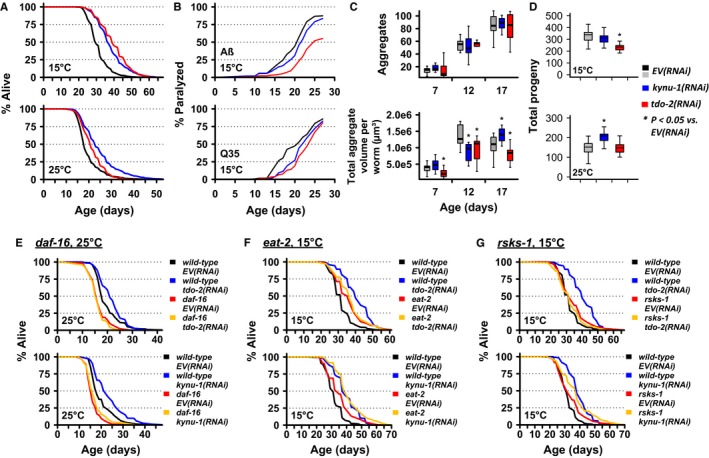
RNAi knockdown of *kynu‐1* and *tdo‐2* produces distinct aging phenotypes. (A) *kynu‐1*(*RNAi*) or *tdo‐2*(*RNAi*) robustly extends lifespan at 15 and 25 °C and (B) delays pathology in worms expressing amyloid‐beta (Aβ; modeling Alzheimer's disease) or a 35‐unit polyglutamine repeat (Q35; modeling Huntington's disease) in body wall muscle at 15 °C. (C) Neither *kynu‐1*(*RNAi*) nor *tdo‐2*(*RNAi*) affects the number of Q35::YFP aggregates that accumulate with age (top). *tdo‐2*(*RNAi*) significantly reduces total Q35::YFP volume per worm at all ages, while *kynu‐1*(*RNAi*) only does so in middle‐aged worms. *P < 0.05 vs. *EV*(*RNAi*) (Student's *t*‐test). (D) *tdo‐2*(*RNAi*) significantly reduces brood size at 15 °C, while *kynu‐1*(*RNAi*) significantly increases brood size at 25 °C. *P < 0.05 vs. *EV*(*RNAi*) (Student's *t*‐test). *kynu‐1*(*RNAi*), but not *tdo‐2*(*RNAi*), extends lifespan of (E) *daf‐16* mutants at 25 °C, (F) *eat‐2* mutants at 15 °C, and (G) *rsks‐1* mutants at 15 °C. For box and whisker plots, center line indicates median, boxes indicate 25th and 75th percentiles, and whiskers indicate 5th and 95th percentiles.

*kynu‐1*(*RNAi*) and *tdo‐2*(*RNAi*) behaved similarly in several respects, robustly extending lifespan at both 15 and 25 °C (Fig. 5A; Table S12, Supporting information), extending all three measures of healthspan at 15 and 25 °C (Fig. S9; Table S9, Supporting information), and delaying paralysis in Aβ worms at 15 and 25 °C and in Q35 worms at 15 °C alone (Figs 5B and S6B; Table S10, Supporting information). Neither gene affected the number of aggregates that accumulated with age, but *tdo‐2*(*RNAi*) reduced total aggregate volume at all ages, while *kynu‐1*(*RNAi*) reduced aggregate volume only in middle‐aged worms (Fig. 5C). Starker differences became apparent when we examined reproduction and genetic interaction with established aging pathways. We confirmed the protracted reproductive period reported by van der Goot *et al*. (2012) at both temperatures in response to *tdo‐2*(*RNAi*) (Fig. S5B,C, Supporting information) and observed a reduction in total brood size at 15 °C (Fig. 5D; Table S8, Supporting information). In contrast, *kynu‐1*(*RNAi*) increased brood size at 25 °C, but did not otherwise substantively alter the reproductive profile (Figs 5D and S5B,C, Supporting information). As expected from prior studies (e.g. Gems *et al*., 1998; Petrella, 2014), brood size was markedly lower for all worms at 25 than 15 °C (Fig. 5D). Similar to 25 °C, *kynu‐1*(*RNAi*) significantly extended lifespan in all aging mutants at 15 °C (Fig. S7B; Table S11, Supporting information), while lifespan extension from *tdo‐2*(*RNAi*) was eliminated in *daf‐16* mutants at 25 °C, in *eat‐2* mutants at 15 °C, and in *rsks‐1* mutants at 15 °C (Fig. 5E–G; Table S11, Supporting information). As van der Goot *et al*. (2012) found lifespan extension from *tdo‐2*(*RNAi*) to be dependent on *daf‐16* and independent of *eat‐2* at 20 °C, the environmental context at 20 °C is likely more similar to 25 °C than 15 °C.

In summary, we were largely able to confirm the observations of van der Goot *et al*. (2012) and conclude that (i) the mechanisms linking *tdo‐2* to aging are strongly temperature dependent, and (ii) *kynu‐1*(*RNAi*) and *tdo‐2*(*RNAi*) have broadly similar effects on aging phenotypes, but achieve these outcomes through distinct mechanisms.

## Discussion

In this study, we screened 82 *C. elegans* orthologs of 61 human genes that are differentially expressed with age in human blood. We report significant lifespan extension in response to RNAi knockdown of 40 of these genes. The CHARGE gene set was markedly enriched for genes capable of directly altering lifespan, suggesting a high degree of evolutionary conservation in genes influencing aging. Of the 40 pro‐aging genes in the CHARGE gene set and an additional 10 identified in the Random gene set, only four have been previously reported, while the remaining 46 are novel.

The percentage of candidate genes that directly affected lifespan at 25 °C (43.9%) was substantially higher than RNAi screens in *C. elegans* without a preselection criteria (<2%) (Lee *et al*., 2003b; Hamilton *et al*., 2005; Hansen *et al*., 2005; Samuelson *et al*., 2007) and within the range for screens that preselected candidates based on secondary *C. elegans* phenotypes, including developmental arrest (2–42%) (Chen *et al*., 2007; Curran & Ruvkun, 2007), reproductive senescence (16%) (Wang *et al*., 2014), thermal stress resistance (78%) (Munoz & Riddle, 2003), oxidative stress resistance (13–67%) (de Castro *et al*., 2004; Kim & Sun, 2007), and activation of the mitochondrial unfolded‐protein response (53%) (Bennett *et al*., 2014) (reviewed by Yanos *et al*., 2012 and Sutphin & Korstanje, 2016). Tacutu *et al*. (2012) found that knockdown of 27% of *C. elegans* orthologs of genes found in human or worm longevity protein–protein interaction networks increased lifespan. While differences in strain, environment, and criteria for identifying long‐lived candidates make comparison between screens necessarily qualitative, it is remarkable that preselection of *C. elegans* orthologs of human genes differentially expressed with age resulted in a similar enrichment for longevity determinants as preselection based on secondary phenotypes known to correlate with *C. elegans* longevity. Our results validate the use of *C. elegans* as a platform to directly screen human candidate aging genes identified through the application of ‘‐omics’ technologies and rapidly prioritize candidates for further study.

### Environmental temperature is a key regulator of longevity in *C. elegans*

Temperature is consistently identified as a critical environmental factor in *C. elegans* aging. Previous work has detailed the role of temperature in specific aging interventions, such as reduced insulin signaling (Gems *et al*., 1998), reduced hypoxic signaling (Leiser *et al*., 2011), caffeine supplementation (Sutphin *et al*., 2012), or disruption of physiological ‘clock’ genes (Lakowski & Hekimi, 1996), among others. Lee & Kenyon (2009) and Horikawa *et al*. (2015) identified the steroid receptor, *daf‐12*, and p23 cochaperone, *daf‐41*, as mediators of the influence neurosensory machinery on temperature‐specific longevity. Zhang *et al*. (2015) defined the critical life stages during which temperature influences longevity and identified a thermosensitive TRP channel as a mediator of this influence. These studies combined with our observed lack of correlation between lifespan extension at 15 and 25 °C in the CHARGE gene set (Fig. 2C) suggest that different sets of processes drive aging in each environmental context. For instance, we found *tdo‐2*(*RNAi*) lifespan extension to require *daf‐16*, but not *rsks‐1,* at 25 °C, and require *rsks‐1*, but not *daf‐16*, at 15 °C (Fig. 5E,G). *Caenorhabditis elegans* aging studies at 15 °C are relatively rare compared to studies at 20 and 25 °C, and we anticipate that future studies at 15 °C will uncover novel molecular processes capable of influencing aging.

The observed temperature‐specific enrichment for aging genes suggests that the environmental context experienced by aging human blood cells is more analogous to the physiological state of *C. elegans* at 25 than 15 °C. Similar screens using age‐associated expression data from other tissues will provide insight into whether this temperature specificity is a general or tissue‐specific phenomenon. Alternatively, 25 °C may present a lower hurdle to lifespan extension, with a nearly twofold lower mean lifespan for wild‐type worms than at 15 °C. However, our observation that a similar fraction of RNAi are capable of increasing lifespan at 15 and 25 °C in the Random gene set suggests that this is not the case. A growing body of work has begun to define the genetic landscape underlying the response of aging worms to environmental temperature. To what extent gene–environment interactions can be attributed to specific mechanisms, such as differences in metabolic rate or exposure to environmental stressors, will require further study.

### Interaction with established aging pathways prioritizes candidates

In further examining the five genes with the greatest impact of lifespan, we found distinct interactions between each candidate gene and established aging pathways. While interaction data alone are insufficient to definitively identify molecular mechanisms, examining these interactions in the context of published work helps determine which genes are of interest for detailed follow‐up studies:

#### TSP‐3 and the hypoxic response

TSP‐3 is a *C. elegans* tetraspanin, a family of transmembrane scaffolding proteins involved in cell adhesion, motility, activation, and proliferation. We observed that *tsp‐3*(*RNAi*) lifespan extension is prevented by deletion of either *hif‐1* or *daf‐16* (Fig. 4B). DAF‐16 is also required for lifespan extension resulting from deletion of *hif‐1* at 25 °C (Leiser *et al*., 2011), suggesting that knockdown of *tsp‐3* may extend lifespan by HIF‐1‐mediated activation of DAF‐16. Expression of human *TSPAN13* is downregulated both with age (Peters *et al*., 2015) and in breast cancers (Huang *et al*., 2005). Human HIF‐1α and HIF‐2α are upregulated in many cancers, promoting a procancer transcriptional program (Semenza, 2010). Age‐dependent loss of *TSPAN13* may contribute to cancer risk by elevating HIF‐1‐mediated transcription. Tetraspanins and their link to hypoxic signaling will be an interesting topic for future study in the context of longevity and cancer.

#### RCAN‐1 as a context‐dependent regulator of calcineurin (RCN)

RCAN‐1 regulates calcineurin, a calcium‐ and calmodulin‐dependent serine/threonine phosphatase that modulates cellular growth, survival, and inflammation. We find that lifespan extension from *rcan‐1*(*RNAi*) was eliminated in *eat‐2* mutants and suppressed in *rsks‐1* mutants (Fig. 4C), suggesting that *rcan‐1*(*RNAi*) increases lifespan by inhibiting mTOR signaling in a manner similar to DR. In support of this model, increased autophagy resulting from calcineurin inhibition in rat cardiomyocytes is mediated by mTOR through decreased S6K activity (He *et al*., 2014). RCAN‐1 is typically studied as an inhibitor of calcineurin (Lee *et al*., 2003a), implying that *increased* calcineurin activity should promote longevity. However, *reduced* calcineurin activity is reported to increase worm lifespan, mediated by increased autophagy (Dwivedi *et al*., 2009). Studies reporting calcineurin inhibition by RCNs have often been conducted in the context of RCN overexpression, while in the normal range of physiological expression human and yeast RCN can either activate or inhibit calcineurin, depending on context (Hilioti *et al*., 2004). This suggests that *C. elegans* at 25 °C may be in a physiological state where RCAN‐1 *activates* calcineurin, and *rcan‐1*(*RNAi*) knockdown extends lifespan by reducing activity through calcineurin and mTOR. Clarifying the complex regulatory relationship between *rcan‐1* and calcineurin may open the way to new interventions designed to regulate autophagy by modulating RCN activity in specific physiological contexts.

#### Kynurenine metabolism offers multiple, distinct aging intervention targets

Among the five genes examined, only *kynu‐1*(*RNAi*) extended lifespan at both 15 and 25 °C, increased lifespan in all aging pathway mutants, and delayed pathology in the Alzheimer's and Huntington's disease models. Inhibition of *kynu‐1* influenced aging in a manner distinct from inhibition of the previously studied kynurenine pathway gene, *tdo‐2*. Specifically, RNAi targeting each gene resulted in distinct reproductive patterns, different effects on age‐dependent Q35 aggregation, and distinct interaction with established aging pathways in the context of lifespan.

The structure of the kynurenine pathway suggests that the observed phenotypic differences are mediated through differential influence on kynurenine metabolites. The kynurenine pathway is the primary metabolic destination for TRP. TDO catalyzes the initial enzymatic step, resulting in the conversion of TRP to KYN. KYN is then metabolized through two downstream branches: The *NAD branch* converts KYN to nicotinamide adenine dinucleotide (NAD) through a series of intermediate enzymatic steps, while the *KYNA branch* converts KYN to kynurenic acid (KYNA), catalyzed by kynurenine aminotransferases. While *tdo‐2* mediates the initial entry of TRP into the pathway, KYNU/*kynu‐1* falls downstream of KYN in the NAD branch. Knockdown of *tdo‐2* is thought to influence *C. elegans* aging by increasing TRP concentrations (van der Goot *et al*., 2012). Similar to *tdo‐2*(*RNAi*), adding TRP to the culture media activates DAF‐16 reporters and extends lifespan of wild‐type worms in a manner that is nonadditive with *eat‐2* mutation (Edwards *et al*., 2015).

Worms lacking *kynu‐1* do have slightly increased TRP, but substantially less than animals fed *tdo‐2*(*RNAi*) (van der Goot *et al*., 2012), indicating that *kynu‐1* aging phenotypes are likely TRP independent. Instead, inhibition of *kynu‐1* increases KYN levels and may influence aging by increasing activity through the KYNA branch of the pathway. KYNA inhibits NMDA and α7 nicotinic acetylcholine receptors and can act as an antioxidant, providing potential mechanisms of action. Interventions that increase physiological KYNA levels attenuate neurodegeneration in mouse and fly models of Huntington's disease (Schwarcz *et al*., 2012). Alternatively, the influence of *kynu‐1* on aging may be mediated by increased concentration of 3HK, the metabolite immediately upstream of *kynu‐1* in the kynurenine pathway (van der Goot *et al*., 2012). 3HK has complex pro‐ and antioxidant properties and can also be converted to xanthurenic acid (XA) by KAT enzymes. XA is thought to impair the production, release, and activity of insulin (Oxenkrug, 2013). Increased XA production may explain a partial repression of *kynu‐1*(*RNAi*) lifespan extension by deletion of *daf‐16* that we observed at 25 °C (Fig. 5E).

Altered kynurenine pathway metabolism is linked to many human diseases of aging, including neurodegeneration (Schwarcz *et al*., 2012), chronic inflammation (Oxenkrug, 2011), and diabetes (Oxenkrug, 2013). Kynurenine enzymes are being pursued as clinical targets for neurodegenerative and inflammatory disease, with at least two studies reporting that pharmacological inhibitors improve cognition (Pocivavsek *et al*., 2011; Zwilling *et al*., 2011). Mechanistic differences between branches of the kynurenine pathway are directly relevant to age‐associated disease in humans, where the dominant pathway branch varies by cell and tissue type (Schwarcz *et al*., 2012). While kynurenine metabolism is an active area of interest for many types of age‐associated disease, *KYNU*/*kynu‐1* is one of the least studied targets. Our work highlights KYNU as an intriguing target with the potential to produce distinct clinical outcomes compared to more commonly studied kynurenine pathway enzymes.

#### iglr‐1 interacts with multiple aging pathways

The highly conserved NLRR proteins are involved in organizing neural connectivity, particularly during development. The human gene that led to the selection of *iglr‐1*,* LRRN3,* is consistently found to be among the most downregulated with age in human blood (Hong *et al*., 2008; Harries *et al*., 2011; Marttila *et al*., 2013). Lifespan extension from *iglr‐1*(*RNAi*) was eliminated in each aging mutant examined except *sir‐2.1* (Fig. 4A), indicating a complex interaction with multiple aging pathways. The consistent age‐associated downregulation in humans makes *LRRN3*/*iglr‐1* an interesting candidate, but additional characterization is needed to identify a specific direction for mechanistic follow‐up studies.

#### Lifespan extension via unc‐36 inhibition is worm specific

UNC‐36 is a voltage‐gated calcium channel involved in muscle calcium signaling. Similar to *rcan‐1*, RNAi knockdown of *unc‐36* failed to extend lifespan in either *eat‐2* or *rsks‐1* worms (Fig. 4D). The link between *unc‐36* and the DR/mTOR pathway is straightforward. Worms with loss‐of‐function mutations in *unc‐36* are known to have reduced pharyngeal pumping, limiting food intake (Avery, 1993) and resulting in a genetic mimetic of DR similar to *eat‐2* mutation. Mimicking DR via reduced pharyngeal pumping is clearly a worm‐specific phenomenon, placing *unc‐36* at a low priority for further study.

## Conclusions and future directions

This study highlights the strength of *C. elegans* as a comparative tool for prioritizing human candidate aging genes and confirms age‐associated gene expression as a rich source for novel genes that play a causative role in aging. The temperature‐dependent enrichment for longevity determinants and the temperature‐specific interactions observed for specific candidate genes emphasize the importance of environmental context in mechanistic studies of *C. elegans* aging. Our results identify many novel aging genes and specifically promote *kynu‐1*,* tsp‐3*, and *rcan‐1* as priority candidates for detailed mechanistic follow‐up.

## Experimental procedures

### Worm culture

We maintained worms on solid nematode growth media (NGM) seeded with UV‐killed *Escherichia coli* (OP50) bacteria at 20 °C as described (Sutphin & Kaeberlein, 2009). We conducted RNAi feeding, lifespan, healthspan, brood size, paralysis, and aggregate quantification assays according to standard protocols.

### Candidate gene selection

We constructed candidate gene sets by selecting the 125 genes with the lowest *P*‐values in the whole blood expression meta‐analysis from the CHARGE study (Peters *et al*., 2015) (CHARGE gene set) or by randomly selecting genes from the complete Ensembl human genome (v77, www.ensembl.org) (Random gene set). We used the WORMHOLE ortholog prediction tool (wormhole.jax.org; Sutphin *et al*. (2016)) to identify *C. elegans* orthologs for each human candidate and obtained RNAi clones from the Ahringer or Vidal *C. elegans* RNAi feeding libraries (Table S13, Supporting information).

### Lifespan screen

We conducted lifespan screening in two tiers, first measuring the effect of each candidate RNAi on lifespan at 15 and 25 °C. Any RNAi that significantly extended lifespan relative to experiment‐matched *EV*(*RNAi*) was validated in at least two additional rounds of lifespan measurement.

Appendix S1: Experimental Procedures provides additional detail for all methods.

## Funding

This work was supported by the National Institute on Aging grant AG038070 and the National Cancer Institute Core grant CA034196 to The Jackson Laboratory. MJP and JBJM were supported by the Netherlands Organization for Scientific Research (NWO) VIDI grant 917103521. ADJ purchased worm screening libraries and was supported by National Heart, Lung, and Blood Institute Intramural Funds.

## Author contributions

GS, JMM, ADJ, and RK conceived of the study and planned experiments. GS, GB, SS, SB, CC, and TL carried out experiments. All authors analyzed and interpreted data. GS prepared the manuscript. GS, GB, SS, MJP, JBJM, JMM, ADJ, and RK contributed to writing and critically reviewed the manuscript.

## Conflict of interest

None declared.

## Supporting information


**Fig. S1** RNAi knockdown of 5 out of 82 genes in CHARGE gene set increases lifespan at 15 °C.
**Fig. S2** Lifespan screen survival curves for the CHARGE gene set.
**Fig. S3** Lifespan screen survival curves for the Random gene set.
**Fig. S4** Knockout mutants validate RNAi lifespan phenotypes.
**Fig. S5** Number of eggs produced per day.
**Fig. S6** Impact of candidate RNAi on pathology in *C. elegans* Alzheimer's (Aβ) and Huntington's (Q35) disease models.
**Fig. S7** Lifespan genetic interaction survival curves.
**Fig. S8** RNAi targeting kynurenine pathway genes produces expected metabolic response.
**Fig. S9** Kynurenine pathway inhibition extends healthspan.

**Table S1** 125 human genes most significantly differentially expressed in CHARGE aging study.

**Table S2** *C. elegans* orthologs predicted based on CHARGE human candidate genes.

**Table S3** *C. elegans* orthologs predicted based on randomly selected human genes.

**Table S4** Summary statistics for *C. elegans* lifespan screen (CHARGE gene set).

**Table S5** Summary statistics for *C. elegans* lifespan screen (Random gene set).

**Table S6** Summary of linear mixed models examining the effect of gene set on lifespan when target genes are knocked down using RNAi in *C. elegans*.

**Table S7** Summary statistics for null mutant lifespan experiments.

**Table S8** Summary statistics for brood size experiments.

**Table S9** Summary statistics for healthspan experiments.

**Table S10** Summary statistics for paralysis experiments in *C. elegans* Alzheimer's (Aβ) and Huntington's (Q35) disease models.

**Table S11** Summary statistics for lifespan genetic interaction experiments.

**Table S12** Summary statistics for kynurenine pathway lifespan experiments.

**Table S13** *C. elegans* RNAi feeding library clones used in this study.

**Appendix S1** Experimental procedures.
