# Supplementary material for: Caenorhabditis elegans orthologs of human genes differentially expressed with age are enriched for determinants of longevity
Source: Aging Cell. 2017 Apr 12;16(4):672–82. doi: 10.1111/acel.12595 (PMC5506438; doi:10.1111/acel.12595)

Fig S1. RNAi knockdown of 5 out of 82 genes in CHARGE gene set increases lifespan at 15°C. Bars represent percent change in mean lifespan for RNAi targeting each gene in the CHARGE gene set relative to experiment-matched *EV(RNAi)* when experiments are pooled. Error bars indicate standard error. Long-lived genes based on our screen criteria are colored blue. For each *C. elegans* gene included in the screen, the table to the left indicates the corresponding human ortholog, the ortholog confidence category (high-confidence ortholog, HCO; related protein, RP), whether RNAi was applied staring at egg or at the L4 larval stage, and the direction of change in expression of each human gene with age in the specified tissues (lymphoblastoid cell line, LCL; peripheral blood mononuclear cell, PBMC). Human expression data was assembled from Peters et al. (2015).

Worm Gene	Human Gene	Ortholog Category	RNAi Stage	Change in Human Gene Expression with Age								
				Whole Blood	Cerebellum	Frontal Cortex	CD4+ T Cells	CD8+ T Cells	Monocytes	LCLs	Lymphocytes	PBMCs
kynu−1	***KYNU***	**HCO**	**egg**	**+**	**?**	**?**	**n**	**+**	**n**	**n**	**?**	**n**
T22D1.3	*IMPDH2*	HCO	egg	−	n	n	n	−	n	n	?	n
kel−1	*KLHL3*	HCO	egg	−	n	n	−	−	n	n	−	−
srp−3	*SERPINE2*	RP	egg	−	−	−	−	−	n	n	?	n
ceeh−2	*EPHX2*	RP	egg	−	n	?	−	−	n	n	?	−
nex−3	*ANXA1*	RP	egg	+	n	n	+	+	n	+	+	n
C31B8.8	*MMP28*	RP	egg	−	?	n	−	−	n	n	?	−
aat−6	*SLC7A6*	RP	egg	−	+	n	−	−	n	+	?	−
lec−2	*LGALS1*	RP	egg	+	n	n	+	+	n	n	?	n
unc−82	*NUAK1*	HCO	egg	+	n	n	n	?	+	?	?	n
aat−4	*SLC7A6*	RP	egg	−	+	n	−	−	n	+	?	−
enpl−1	*HSP90AB1*	RP	egg	−	n	n	n	?	−	n	−	−
ldh−1	*LDHB*	HCO	egg	−	−	−	−	−	−	+	?	n
obr−3	*OSBPL5*	HCO	L4	+	+	n	n	+	n	?	?	n
F19C6.3	*NELL2*	RP	egg	−	n	−	n	?	n	?	−	−
unc−36	***CACNA2D2***	**RP**	**egg**	**+**	**n**	**n**	**+**	**+**	**+**	**n**	**?**	**+**
snt−4	*SYT11*	HCO	egg	+	−	−	+	+	n	n	+	+
atm−1	*ATM*	HCO	egg	−	?	?	n	−	n	−	?	−
F36H2.3	*CR2*	HCO	egg	−	?	?	−	−	n	−	?	−
aat−3	*SLC7A6*	RP	egg	−	+	n	−	−	n	+	?	−
unc−115	*ABLIM1*	HCO	L4	−	n	+	−	−	n	n	?	−
rcan−1	***RCAN3***	**RP**	**egg**	**−**	**?**	**?**	**−**	**−**	**−**	**n**	**−**	**n**
atl−1	*ATM*	RP	egg	−	?	?	n	−	n	−	?	−
spe−8	*TXK*	HCO	egg	−	?	?	−	−	n	+	?	n
dyf−3	*CLUAP1*	HCO	egg	−	n	n	n	n	−	n	?	n
mct−3	*SLC16A10*	RP	egg	−	+	−	−	−	n	+	n	−
aat−2	*SLC7A6*	HCO	egg	−	+	n	−	−	n	+	?	−
D2030.8	*PCED1B*	HCO	L4	−	?	?	n	−	n	+	−	−
srp−2	*SERPINE2*	RP	egg	−	−	−	−	−	n	n	?	n
cup−5	*MCOLN2*	HCO	egg	+	?	?	+	+	n	−	?	n
sem−5	*GRAP*	HCO	egg	−	?	?	−	−	n	n	?	−
sel−2	*NBEA*	HCO	egg	−	−	−	−	?	−	+	?	−
T13H5.1	*PTPRK*	RP	L4	−	−	+	−	−	n	n	−	−
lron−11	*LRRN3*	RP	egg	−	n	−	−	?	−	n	?	−
try−3	*GZMH*	RP	egg	+	?	?	+	+	+	?	?	n
srp−5	*SERPINE2*	RP	egg	−	−	−	−	−	n	n	?	n
nucb−1	*NUCB2*	HCO	egg	−	−	n	−	−	−	n	?	−
mca−2	*ATP2B4*	RP	L4	+	n	n	+	?	n	n	?	n
T01D3.6	*FCGBP*	HCO	egg	−	?	+	n	−	n	n	?	−
atic−1	*ATIC*	HCO	egg	−	n	+	n	−	n	+	−	n
K01G5.5	*DKC1*	HCO	L4	−	?	?	n	?	n	n	−	−
srp−1	*SERPINE2*	RP	egg	−	−	−	−	−	n	n	?	n
tag−180	*CACNA2D2*	HCO	egg	+	n	n	+	+	+	n	?	+
aat−7	*SLC7A6*	RP	egg	−	+	n	−	−	n	+	?	−
daf−21	*HSP90AB1*	RP	L4	−	n	n	n	?	−	n	−	−
C31C9.2	*PHGDH*	HCO	egg	−	n	+	−	−	n	n	−	−
ser−5	*ADRB2*	RP	egg	+	?	?	+	+	n	n	?	n
C04E6.7	*EPHX2*	RP	egg	−	n	?	−	−	n	n	?	−
fib−1	*FBL*	HCO	L4	−	+	+	−	−	−	n	−	n
B0286.3	*PAICS*	HCO	egg	−	n	+	−	−	n	+	?	−
pop−1	*TCF7*	HCO	L4	−	?	?	n	−	n	−	?	n
M01G12.2	*SERPINE2*	RP	egg	−	−	−	−	−	n	n	?	n
iglr−1	***LRRN3***	**RP**	**egg**	**−**	**n**	**−**	**−**	**?**	**−**	**n**	**?**	**−**
aat−1	*SLC7A6*	RP	egg	−	+	n	−	−	n	+	?	−
sym−3	*FAM102A*	HCO	egg	−	n	+	−	−	n	n	?	−
clec−196	*KLRB1*	RP	egg	−	?	?	−	−	n	n	?	n
aat−8	*SLC7A6*	RP	egg	−	+	n	−	−	n	+	?	−
Y54G11A.4	*TTC38*	HCO	egg	+	n	?	+	+	n	?	+	n
tsp−3	***TSPAN13***	**HCO**	**egg**	**−**	**−**	**−**	**n**	**n**	**n**	**n**	**?**	**n**
F37H8.3	*EPHX2*	HCO	egg	−	n	?	−	−	n	n	?	−
cmk−1	*CAMK4*	RP	egg	−	?	?	−	?	n	n	−	−
rho−1	*RHOC*	RP	L4	+	n	+	+	+	n	n	?	+
srp−6	*SERPINE2*	RP	egg	−	−	−	−	−	n	n	?	n
R02E12.5	*C1orf21*	HCO	egg	+	+	−	+	+	+	n	?	+
rpl−22	*RPL22*	HCO	L4	−	n	−	n	−	n	n	?	−
lec−3	*LGALS1*	RP	egg	+	n	n	+	+	n	n	?	n
T28H10.3	*LGMN*	HCO	egg	−	−	−	−	−	n	−	?	n
sur−6	*PPP2R2B*	HCO	egg	+	−	−	+	+	n	n	?	+
C25F6.7	*ITM2C*	HCO	egg	−	n	n	−	−	n	n	?	−
srp−8	*SERPINE2*	RP	egg	−	−	−	−	−	n	n	?	n
Y54G11A.7	*TTC38*	HCO	egg	+	n	?	+	+	n	?	+	n
daf−16	*FOXO1*	RP	egg	−	n	n	n	−	+	+	?	n
dve−1	*SATB1*	HCO	L4	−	?	?	−	−	−	?	−	−
C30B5.2	*LEPROTL1*	HCO	egg	−	n	n	−	−	−	+	−	n
mca−1	*ATP2B4*	RP	L4	+	n	n	+	?	n	n	?	n
R05G6.4	*NOSIP*	HCO	egg	−	n	n	−	−	n	n	−	−
zyg−12	*HOOK1*	HCO	egg	−	+	n	−	−	n	n	?	−
srp−7	*SERPINE2*	RP	egg	−	−	−	−	−	n	n	?	n
fln−1	*FLNB*	RP	egg	−	n	+	−	−	+	n	−	−
aat−9	*SLC7A6*	RP	egg	−	+	n	−	−	n	+	?	−
hpo−29	*NAA16*	HCO	egg	−	n	?	−	−	n	?	−	−
nmt−1	*NMT2*	HCO	L4	−	n	+	−	n	−	−	−	−

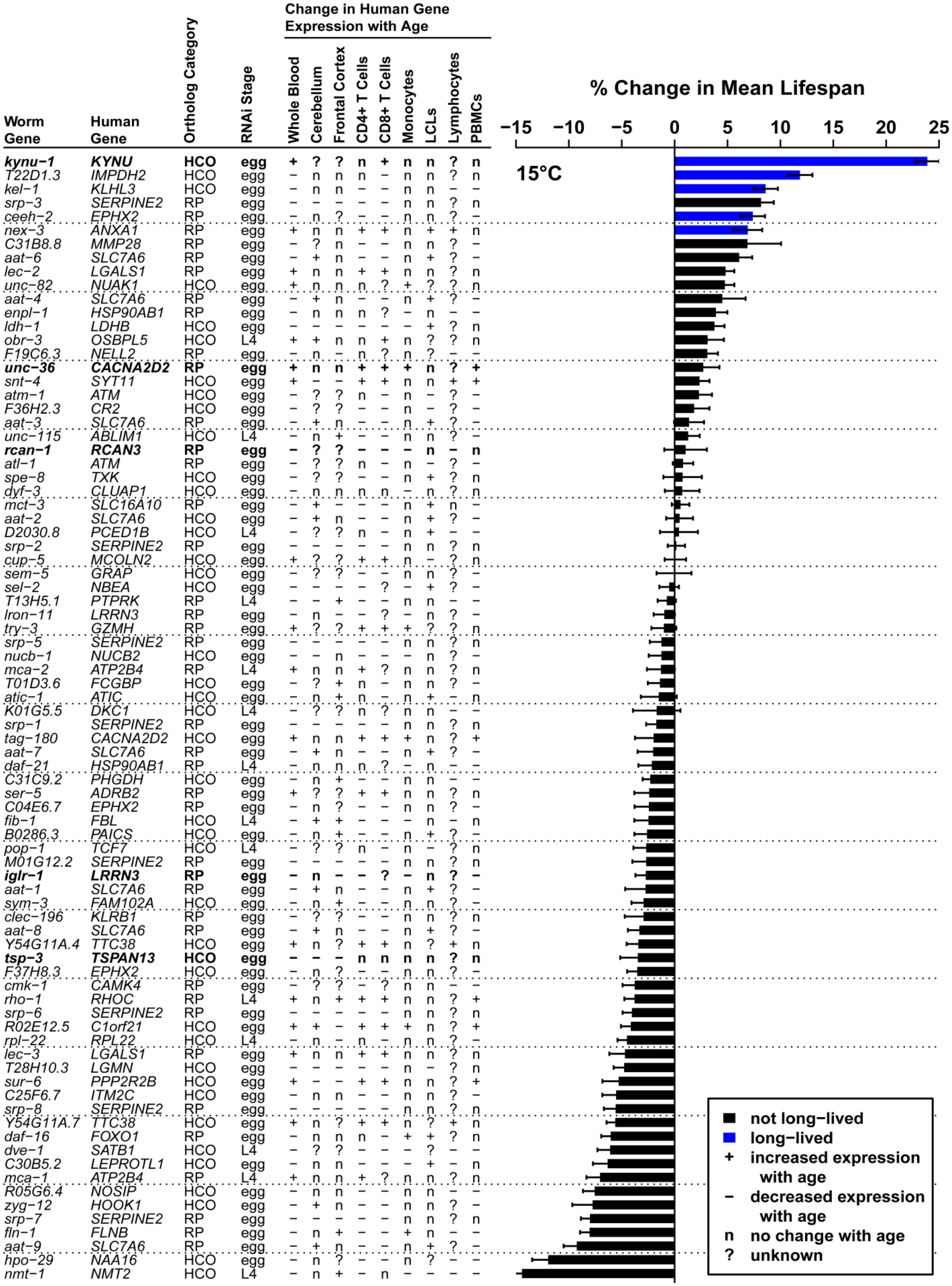

Fig S2. Lifespan screen survival curves for the CHARGE gene set. Each panel shows survival curves pooled across experiments for RNAi targeting a single gene in the CHARGE gene set and experiment-matched *EV(RNAi)* at both 15°C and 25°C.

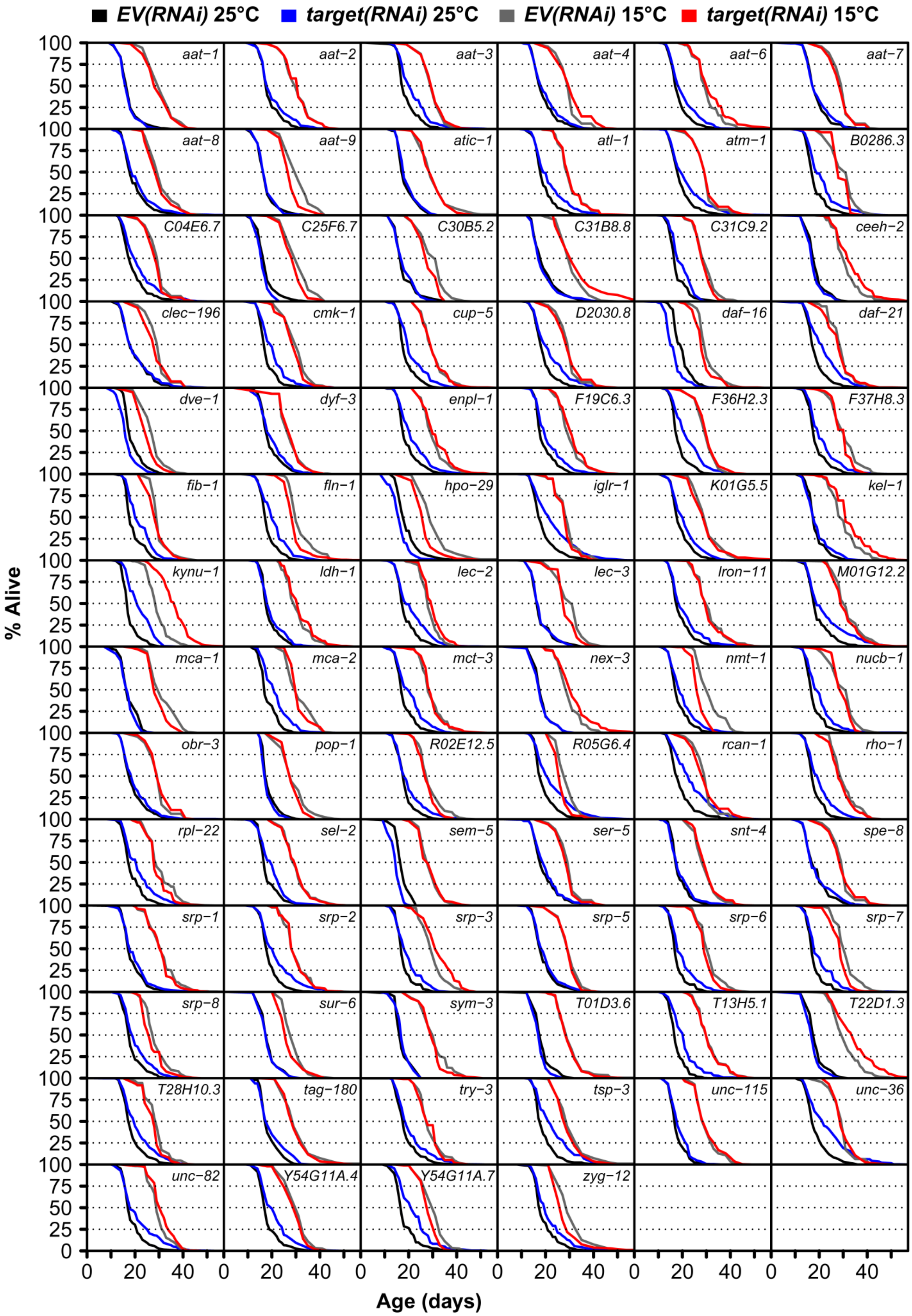

Fig S3. Lifespan screen survival curves for the Random gene set. Each panel shows survival curves pooled across experiments for RNAi targeting a single gene in the Random gene set and experiment-matched *EV(RNAi)* at both 15°C and 25°C.

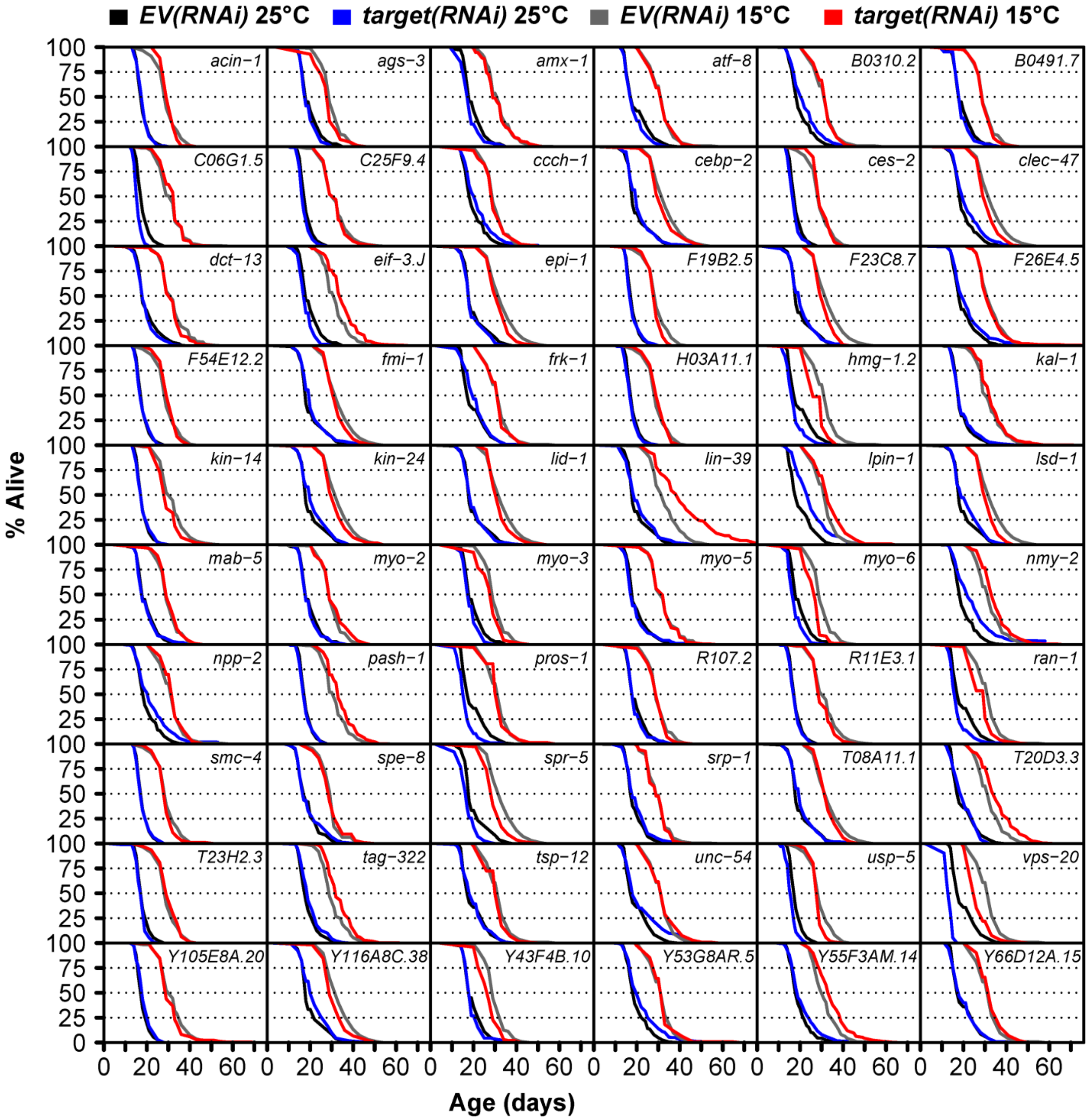

Fig S4. Knockout mutants validate RNAi lifespan phenotypes. Knockout mutants have increased lifespan relative to controls (wild-type worms on *EV(RNAi)*) to a similar degree as wild-type worms on RNAi targeting (**A**) *kynu-1* at 25°C, (**B**) *kynu-1* at 15°C, (**C**) *iglr-1* at 25°C, (**D**) *rcan-1* at 25°C, and (**E**) *tsp-3* at 25°C. (**F**) *unc-36* knockout worms are short-lived relative to controls, while *unc-36(RNAi)* extends lifespan at 25°C. (**G**) Maintaining wild-type worms on *unc-36(RNAi)* for multiple generations partially replicates decreased lifespan of *unc-36* knockout worms relative to wild-type worms maintained on EV*(RNAi)* at 25°C. (**H**) Maintaining wild-type worms on *kynu-1(RNAi)* for multiple generations replicates decreased lifespan extension of *kynu-1* knockout worms relative to wild-type worms maintained on *kynu-1(RNAi)* from egg at 15°C.

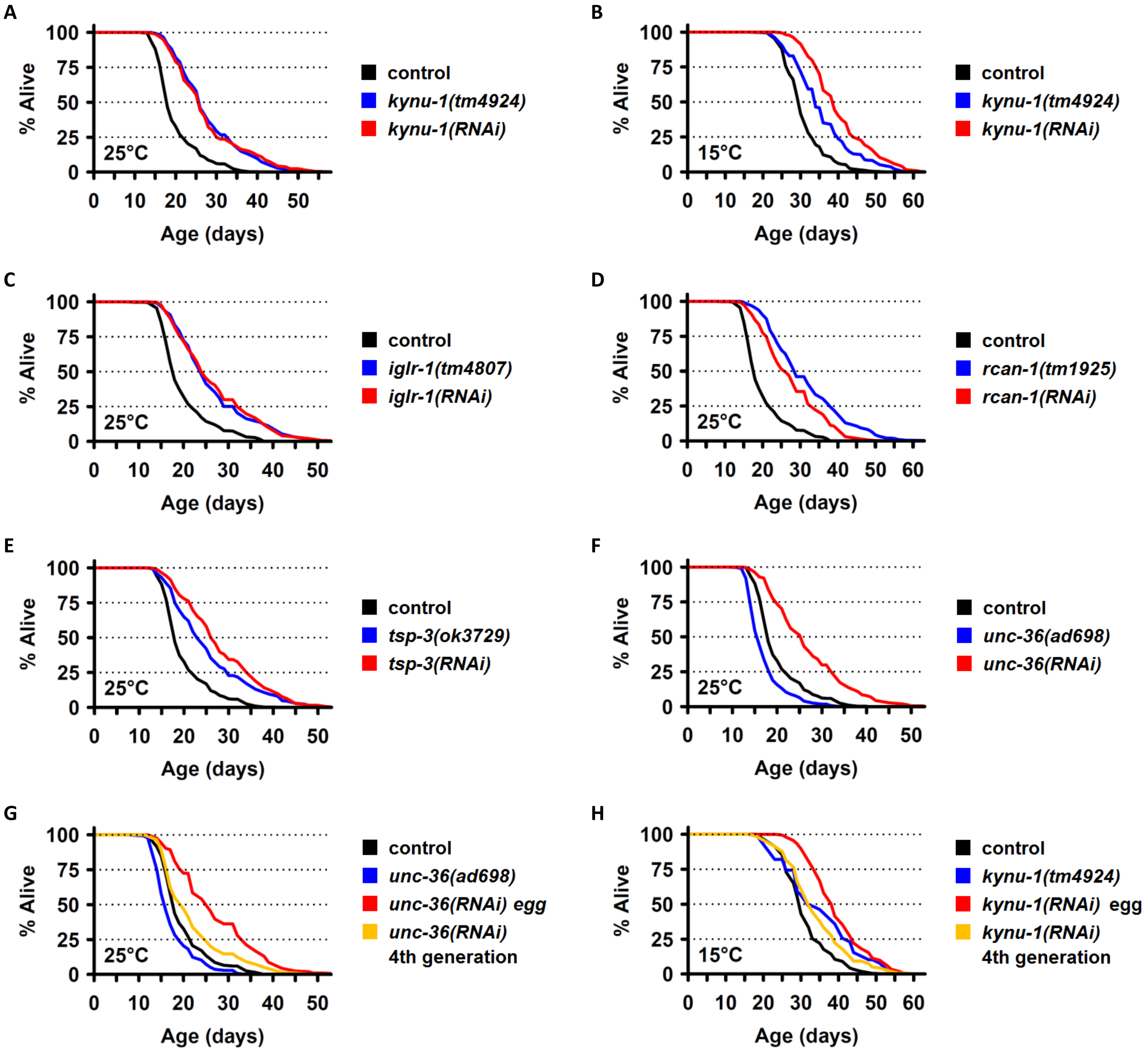

Fig S5. Number of eggs produced per day. (**A**) RNAi knockdown of *iglr-1*, *rcn-1*, or *unc-36* does not affect egg laying, while knockdown of *kynu-1* or *tsp-3* slightly increases the number of eggs laid toward the end of the reproductive period at 25°C. (**B**) *tdo-2(RNAi)* causes a protracted egg-laying period, while *kynu-1(RNAi)* or *haao-1(RNAi)* slightly decreases peak egg-laying at 15°C. (**C**) *tdo-2(RNAi)* causes a protracted egg-laying period, while *kynu-1(RNAi)* or *tsp-3(RNAi)* slightly increases the number of eggs laid toward the end of the reproductive period at 25°C. For box and whisker plots, center line indicates median, boxes indicate 25th and 75th percentiles, and whiskers indicate 5th and 95th percentiles.

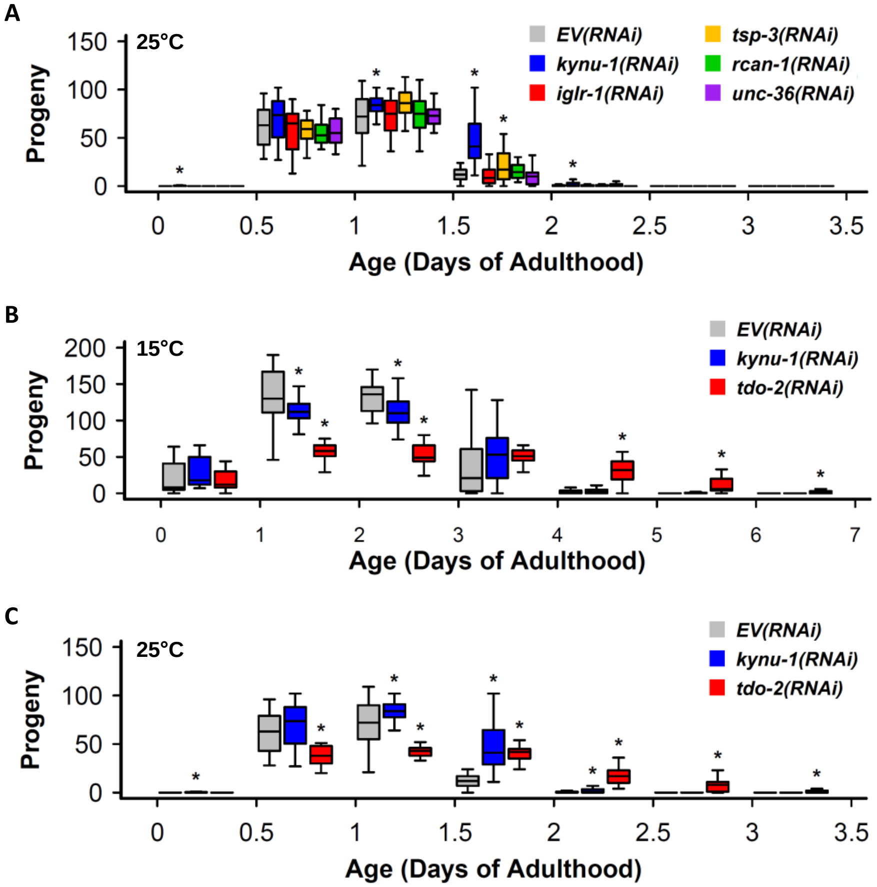

Fig S6. Impact of candidate RNAi on pathology in *C. elegans* Alzheimer's (Aβ) and Huntington's (Q35) disease models. (**A**) RNAi knockdown of *flu-2*, *iglr-1*, *tsp-3*, *rcn-1*, or *unc-36* does not substantially affect age-associated paralysis in worms expressing amyloid-beta (Aβ) or a 35-unit polyglutamine repeat (Q35) in body wall muscle, modeling Alzheimer's or Huntington's disease, respectively, at 25°C. (**B**) RNAi knockdown of *kynu-1* or *tdo-2* slightly delays paralysis in Aβ, but not Q35, worms at 25°C. (**C**) Representative images of3D reconstruction of fluorescently labeled polyglutamine (Q35::YFP) aggregates in young (day 7) and old (day 17) animals subjected to *EV(RNAi)* or *tdo-2(RNAi)*. Left column shows the raw YFP signal for a single image. Right two columns show the automatically reconstructed 3D model with identified aggregates highlighted in color.

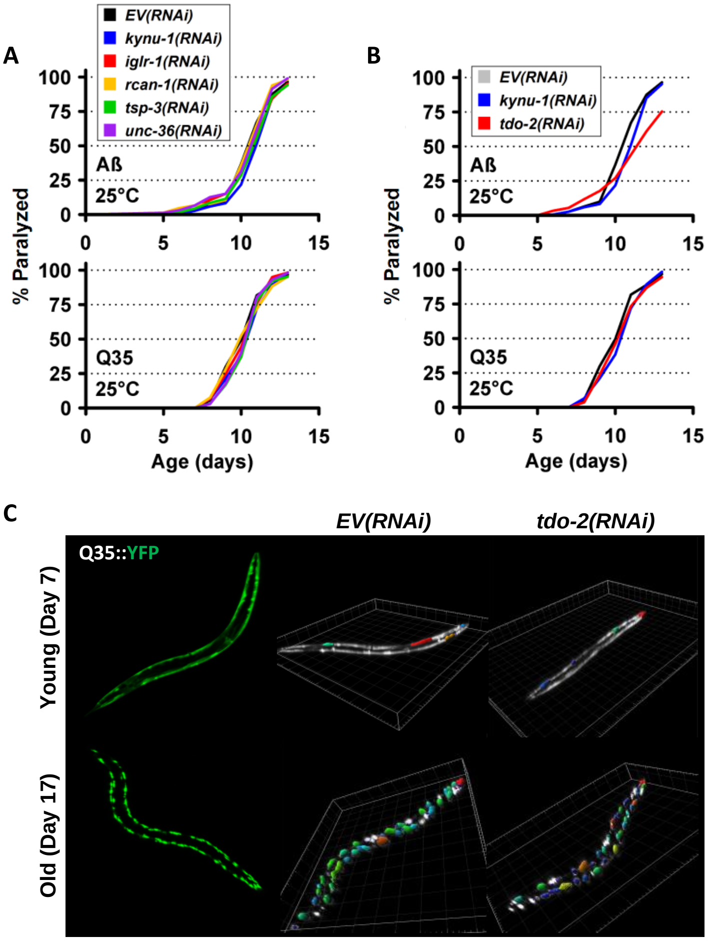

Fig S7. Lifespan genetic interaction survival curves. Each panel shows survival curves pooled across experiments for the interaction between a mutation in a known aging pathway gene and RNAi targeting a candidate gene at (A) 25°C or (B) 15°C. Each panel contains survival curves for aging pathway mutants subjected to the target RNAi and experiment matched wild-type worms subjected to *EV(RNAi)*, wild-type worms subjected to target RNAi, and mutant worms subjected to *EV(RNAi)*.

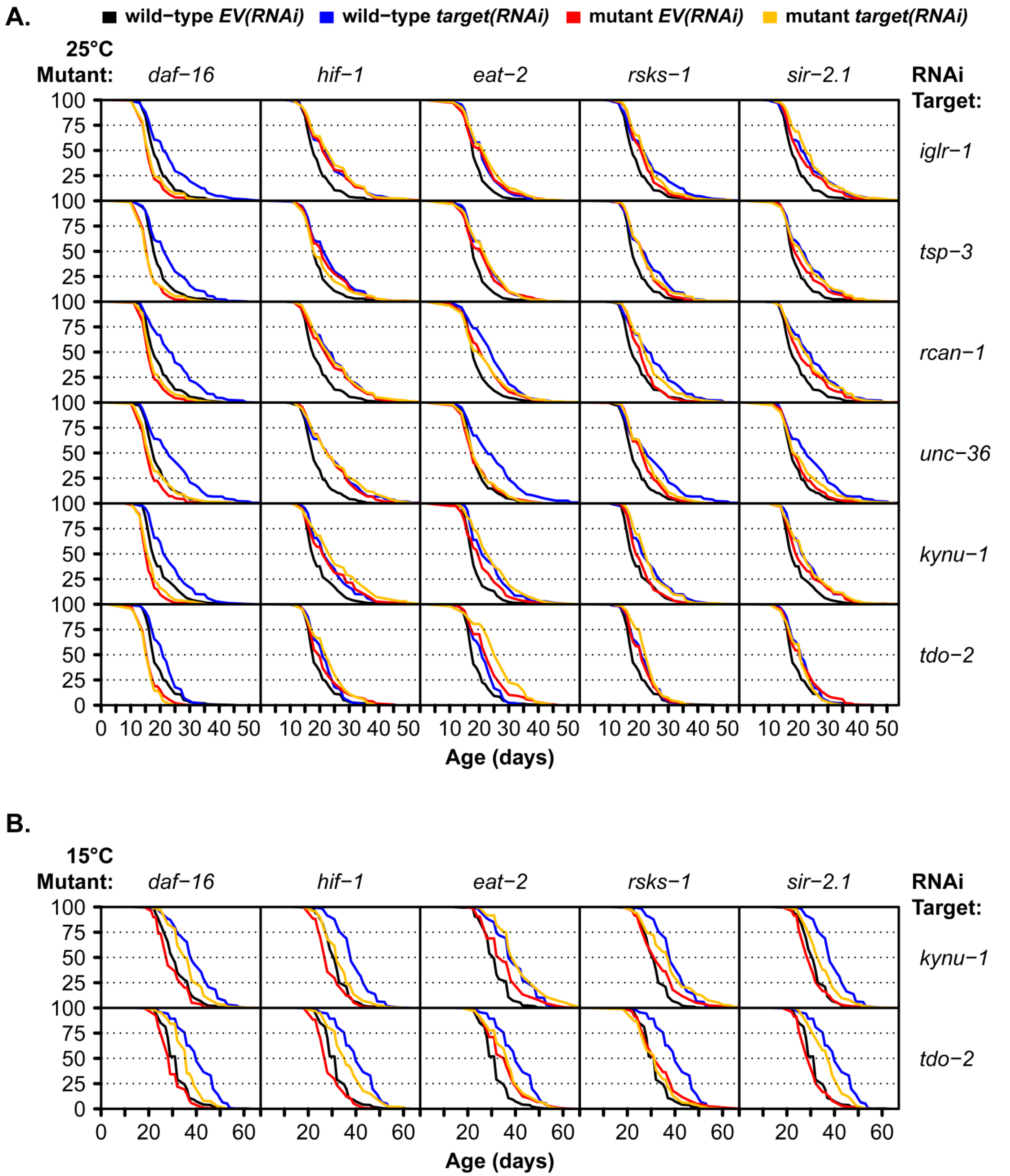

Fig S8. RNAi targeting kynurenine pathway genes produces expected metabolic response. (**A**) Schematic representation of the kynurenine pathway. Measured metabolites are marked with red *s. (**B**) *tdo-2(RNAi)* results in a specific increase in tryptophan, while *kynu-1(RNAi)* results in specific increases in kynurenine and 3-hydroxykynurenine. Bars represent mean metabolite concentration in worms subject to target RNAi relative to empty vector (EV) RNAi. Error bars represent standard error.

A

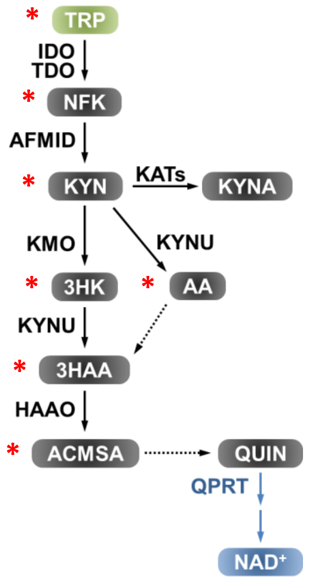

Symbol	Name	Human Gene(s)	Worm Gene(s)
Enzyme Abbreviations:			
TDO	tryptophan 2,3-dioxygenase	*TDO2*	*tdo-2*
IDO	indoleamine 2,3-doxygenase	*IDO1, IDO2*	*n/a*
AFMID	arylformamidase	*AFMID*	*afmd-1, afmd-2*
KAT	kynurenine aminotransferase	*AADAT, CCBL1, CCBL2,*	*nkat-1, nkat-3, tatn-1*
KMO	kynurenine 3-monoxygenase	*KMO*	*kmo-1, kmo-2*
KYNU	kynureninase	*KYNU*	*kynu-1*
HAAO	3-hydroxyanthraniate 3,4-dioxygenase	*HAAO*	*haao-1*
QPRT	quinolinate phosphoribosyl transferase	*Qprt*	*n/a*
Metabolite Abbreviations:			
TRP	tryptophan		
NFK	N-formylkynurenine		
KYN	kynurenine		
KYNA	kynurenic acid		
3HK	3-hydroxykynurenine		
AA	anthranilic acid		
3HAA	3-hydroxyanthranilic acid		
ACMSA	aminocarboxymuconate semialdehyde		
QUIN	quinolinic acid		
NAD^+	nicotinamide adenine dinucleotide		

B

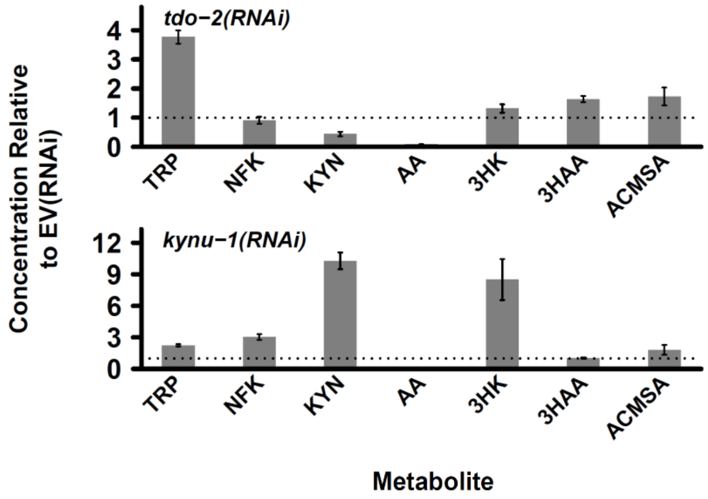

Fig S9. Kynurenine pathway inhibition extends healthspan. RNAi knockdown of *flu-2*, *haao-1*, or *tdo-2* results in a reduction in average speed on solid media when motivate (top) or unmotivated (middle) by plate tapping in young worms that is better maintained throughout lifespan relative to *EV(RNAi)*, while frequency of thrashing in liquid (bottom) is increased in young worms but declines with age at a similar rate to *EV(RNAi)* at both 15°C (**A**) and 25°C (**B**). Each panel presents box plots for each measured parameter for worms subjected to target RNAi at four age points. Data are pooled from three independent experiments, each including 8-10 worms per RNAi. * = P < 0.05 vs. age-matched *EV(RNAi)* (Student's t-test), # = P < 0.05 vs. age-matched *tdo-2(RNAi)* (Student's t-test). For box and whisker plots, center line indicates median, boxes indicate 25th and 75th percentiles, and whiskers indicate 5th and 95th percentiles.

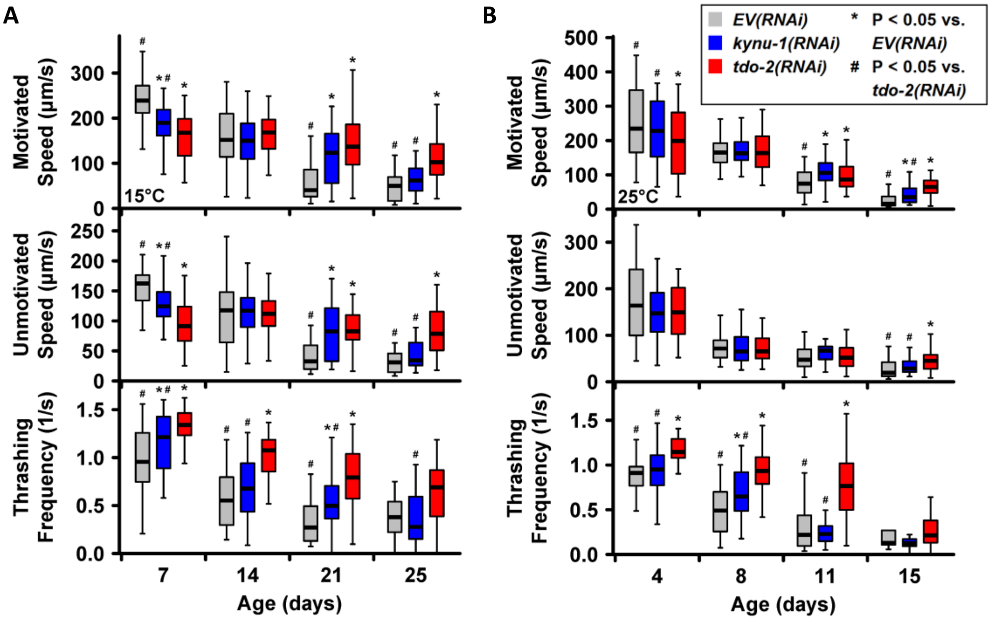

Supplement: Supplementary file 1 — Fig. S1 RNAi knockdown of 5 out of 82 genes in CHARGE gene set increases lifespan at 15 °C. Fig. S2 Lifespan screen survival curves for the CHARGE gene set. Fig. S3 Lifespan screen survival curves for the Random gene set. Fig. S4 Knockout mutants validate RNAi lifespan phenotypes. Fig. S5 Number of eggs produced per day. Fig. S6 Impact of candidate RNAi on pathology in C. elegans Alzheimer's (Aβ) and Huntington's (Q35) disease models. Fig. S7 Lifespan genetic interaction survival curves. Fig. S8 RNAi targeting kynurenine pathway genes produces expected metabolic response. Fig. S9 Kynurenine pathway inhibition extends healthspan. [file ACEL-16-672-s001.pdf]
