# Supplementary material for: Caenorhabditis elegans orthologs of human genes differentially expressed with age are enriched for determinants of longevity
Source: Aging Cell. 2017 Apr 12;16(4):672–82. doi: 10.1111/acel.12595 (PMC5506438; doi:10.1111/acel.12595)
Supplement: Supplementary file 15 — Appendix S1 Experimental procedures. [file ACEL-16-672-s015.pdf]

***C. elegans* Orthologs of Human Genes Differentially Expressed with Age are Enriched for Determinants of Longevity**

Sutphin et al.

SI Experimental Procedures

Worm Strains

The following strains were obtained from the *Caenorhabditis* Genetic Center (CGC) at the College of Biological Sciences at the University of Minnesota: *daf-16(mu86)I* (CF1038), *eat-2(ad465)II* (DA465), *hif-1(ia4)V* (ZG31), *rsks-1(ok1255)III* (RB1206), *sir-2.1(ok434)IV* (VC199), *tsp-3(ok3729)III* (VC3075), *unc-36(ad698)III* (DA698), *unc-54p::Aβ* (CL2006), and *unc-54p::Q35::YFP* (AM140). Strains *iglr-1(tm4807)X* (FX04807), *kynu-1(tm4924)X* (FX04627), and *rcan-1(tm1925)X* (FX01925) were obtained from the *C. elegans* National Bioresource Project (NBRP) at the School of Medicine at the Tokyo Women's Medical University. Wild-type (N2) worms were obtained from Dr. Matt Kaeberlein (University of Washington, Seattle, WA, USA). Strain *rsks-1(ok1255)III* (RB1206) was generated by the *C. elegans* Gene Knockout Project at the Oklahoma Medical Research Foundation as part of the International *C. elegans* Gene Knockout Consortium (Consortium 2012). Strain *sir-2.1(ok434)IV* (VC199) and *tsp-3(ok3729)III* (VC3075) was generated by the *C. elegans* Reverse Genetics Core Facility at the University of British Columbia as part of the International *C. elegans* Gene Knockout Consortium (Consortium 2012).

RNA Interference

All experiments were conducted on NGM containing 1 mM Isopropyl β-D-1-thiogalactopyranoside (IPTG) to activate production of RNAi transcripts and 25 µg/mL carbenicillin to select RNAi plasmids and seeded with live *E. coli* (HT115) containing RNAi feeding plasmids. Worms were age-synchronized via timed egg laying at the experimental temperature and transferred to plates containing 50 µM 5-fluorodeoxyuridine (FUdR) to prevent reproduction at the L4 larval stage as previously described (Sutphin & Kaeberlein 2009).

Lifespan and Paralysis Analysis

Lifespan and paralysis experiments were conducted as previously described (Sutphin & Kaeberlein 2009). Briefly, adult animals were maintained on NGM RNAi plates with FUdR throughout life. Each animal was examined every 1-2 days (25°) or 2-3 days (15°C) by nose- and tail-prodding with a platinum wire pick. For lifespan assays, animals were scored as dead if they failed to react to prodding. Animals were scored as "paralyzed" if they were able to move their head, but were unable to move relative to the plate surface. Live/unparalyzed and dead/paralyzed animals were counted and dead/paralyzed animals removed from the plate. Animals displaying vulva rupture were included in all analyses, while worms that left the surface of the plate were excluded.

Each lifespan and paralysis experiment included 10-16 test groups, each consisting of ~105 worms (3 plates with ~35 worms/plate) subjected to a target RNAi. Each experiment included a negative

control test group fed empty vector (EV) RNAi, and a positive control test group fed RNAi targeting the insulin/IGF receptor gene, *daf-2*, which are consistently long-lived. For lifespan epistasis each combination of candidate gene and aging mutant was measured in three independent experiments, each including: wild type worms fed *EV(RNAi)*, wild-type worms fed candidate RNAi, mutant worms fed *EV(RNAi)*, and mutant worms fed candidate RNAi. P-values for statistical comparison of lifespan between test groups were calculated using the Wilcoxon rank-sum test (*wilcox.test* function in the R "stats" package).

Candidate Gene Selection

We constructed the CHARGE gene set by selecting the 125 genes with the lowest P-values in the whole blood expression meta-analysis from the CHARGE study (Peters *et al.* 2015), identifying *C. elegans* orthologs, obtaining RNAi clones, and confirming the RNAi target sequence. We constructed the Random gene set by randomly selecting genes from the complete Ensembl human genome (v77, www.ensembl.org), identifying *C. elegans* orthologs, obtaining RNAi clones for each target, and confirming the RNAi target sequence. We obtained RNAi clones from the Ahringer or Vidal *C. elegans* RNAi feeding libraries (**Table S13**).

Orthologs were selected using the WORMHOLE ortholog prediction tool (wormhole.jax.org; Sutphin *et al.* (2016)). WORMHOLE uses machine learning to integrate ortholog predictions from 17 ortholog prediction tools and generate a confidence score (the WORMHOLE Score). We defined high-confidence orthologs (HCOs) as orthologs with a WORMHOLE Score ≥ 0.5 and related proteins (RPs) as orthologs with weak consensus among prediction tools (predicted by ≥3 of 17 tools).

Lifespan Screen

We used a two-tiered approach to screen the CHARGE and Random gene sets. In the first tier, lifespan was measured for ~105 worms fed RNAi targeting each gene starting from egg at both 15°C and 25°C. If an RNAi arrested development, the initial round was repeated for that gene with worms maintained on *EV(RNAi)* until the L4 larval stage, then transferred to plates with the target RNAi. In the second tier, each candidate RNAi that significantly extended lifespan relative to experiment-matched *EV(RNAi)* ($p < 0.05$) in the first tier was validated in two additional rounds of lifespan measurement. An additional round was added when one or more experiments had a final worm count of <70 for the candidate or experiment-matched EV RNAi, or to resolve conflicting conclusions from individual experiments. We concluded that a candidate RNAi extended lifespan relative to *EV(RNAi)* if lifespan extension was significant both for data pooled across experiments ($p < 0.05$ with Bonferroni multiple test correction) and for data from two out of three (or three out of four) individual experiments ($p < 0.05$ without multiple test correction). All other worms were considered not long-lived. Researchers were blinded to the gene targets in each experiment. The Wilcoxon Rank-Sum test was used to determine significance between groups in lifespan experiments. Enrichment analysis and the effect of gene set on lifespan were analyzes using Fisher's Exact Test and a log-linear mixed effects model, respectively, as detailed below.

Statistical Methods for Lifespan Screen Analysis

We used Fisher's Exact Test to compare enrichment for lifespan extending RNAi between the CHARGE and Random gene sets. To determine whether worms were significantly longer-lived when subjected to RNAi targeting CHARGE genes relative to Random genes, we built a log-linear mixed effects model for individual lifespan using the *lmer()* function in the "lme4" R package (Bates *et al.* 2015). Gene set was included as a fixed effect with gene and experiment nested within gene as random effects to control for the unbalanced nature of our data (long-lived test groups have more observations than test groups that are not long-lived). Individual lifespan was normalized to mean lifespan of experiment-matched control worms on *EV(RNAi)*. We built separate models for 15°C and 25°C. We used the Satterthwaite method to approximate degrees of freedom and calculate p-values (Satterthwaite 1946).

Kynurenine pathway metabolite quantification

Metabolite extraction. Worms were grown plates containing live *E. coli* (HT115) containing RNAi feeding plasmids targeting *kynu-1(RNAi)* or *tdo-2(RNAi)* at 15°C under conditions identical to those used in the lifespan studies. On day 4 of adulthood, ~100 worms/sample were collected from the plates and washed twice with M9 buffer (21.6 mM Na_2HPO_4 22mM KH_2PO_4 85.6 mM NaCl, 1mM $MgSO_4$). Excess M9 was removed and the worm pellets flash frozen in liquid nitrogen and stored at -80°C prior to metabolite extraction. To extract metabolites, 1 ml of extraction buffer (2:2:1 AcN:MeOH:H2O + internal standards [0.2 ng/ul 1-Napthylamine + 0.2 ng/ul 9-anthracene carboxylic acid]) was added to each sample. Samples were homogenized mechanically and sonicated to dissolve worm pellet and placed at -20°C overnight to precipitate metabolites. Samples were then centrifuged at 20,000 x g for 15 min to pellet protein, and the supernatant transferred to a new tube and dried without heat. Metabolites were resuspended in a solution of 10% acetonitrile in water, both Optima® grade for mass spectrometry analysis.

Relative quantification via mass spectrometry (MS). Samples were run on an Agilent 6530 Q-TOF equipped with a microflow liquid chromatography (LC) system. Separation by reverse-phase LC (RPLC) with a C18 column (Agilent Poroshell, 2.1mm x 50mm) over a 20 minute gradient resulted in separate peaks for each analyte. Detection was in positive ion mode over a mass range of 200-1700 m/z with internal reference ions. Collision-induced dissociation (CID) was employed to confirm the identification of each analyte by mass and retention time. For preliminary analysis, both automated detection and fragmentation of all analytes, as well as detection and fragmentation of targeted analytes were employed. Both resulted in sufficient fragmentation for identification and sufficient intensity for quantification. Subsequent analysis employed quantitative scans for confirmation of relative abundance.

Data analysis. Samples were extracted for target ions using ACD Labs MSWorkbookSuite IntelliTarget feature (http://www.acdlabs.com/products/spectrus/workbooks/ms/msworkbooksuite/). Briefly, a database for the targets of interest was generated and populated using standard compounds as well as library reference data. Targets were extracted and quantified with normalization against an internal standard. Scans were verified manually at both the first and second stages of MS (MS1 and MS2).

Brood size

To measure brood size, 10 worms at the L4 larval stage were placed on individual NGM RNAi plates lacking FUdR and allowed to lay eggs. Worms were transferred to a new plate every 12 hours at 25°C and every 24 hours at 15°C until worms ceased laying eggs. The number of progeny was counted on each plate following a 2 day incubation to allow eggs to hatch. Student's t-tests were used to determine significance in observed differences between target and EV RNAi at each age, and between total progeny counts.

Healthspan

Healthspan data was collected in three independent experiments using the WormLab system (Version 3.1, MBF Bioscience, Williston, VT) at 7, 14, 21, and 25 days of age (15°C) and 4, 8, 11, and 15 days of age (25°C). For speed assays, videos were captured for worms directly on NGM experiment plates. Worms were motivated to begin moving by dropping the plate onto the WormLab video capture stage from a height of ~0.5 inches. Video capture was started immediately and allowed to run for 1 minute, 15 seconds. Worms reacted to the plate drop by moving rapidly for approximately 20 seconds then returning to a normal foraging behavior. For "motivated speed", worms were tracked using the WormLab software for the first 15 seconds of each video. Worms present for at least 14 seconds were selected for analysis. For "unmotivated speed", worms were tracked between 30 seconds and 1 minute, 15 seconds. Worms present for at least 30 seconds were selected for analysis. For thrashing, worms were suspended in a droplet of M9 buffer on unseeded NGM plates and video captured for 45 seconds. Worms present for at least 15 seconds were selected for analysis. Average speed, thrash frequency, body length, and body width for each worm was exported from the WormLab software to R for analysis. The WormLab software tended to misidentify dark sections of the plate as worms. To filter these errors, we initially removed any identified object that fell outside of 1.2 standard deviations in worm body length, width, or area. Based on manual inspection, this threshold removed non-worm objects and poorly identified worms without removing correctly identified worms. Student's t-tests were used to determine significance in differences in each phenotype between target and EV RNAi at each age.

Polyglutamine Aggregate Imaging

We quantified aggregate number and volume using 3D fluorescence microscopy for transgenic worms expressing fluorescently-tagged polyglutamine repeats (Q35::YFP) at 7, 12, and 17 days of age at 15°C. At least 8 animals were examined for each RNAi at each age. Worms were immobilized using 25 nM sodium azide in M9 buffer on microscope slides with 6% agarose pads. Worms were imaged using a Leica TCS SP8 Laser Scanning Microscope equipped with a 10x objective. Z-stack images were collected for whole worms with stack spacing selected based on the Nyquist sampling theorem (minimum 2.3 images per minimum aggregate diameter). The microscope was set to capture images with resonance scanning and line averaging of 16. Images for worms that did not fit a single field of view were merged with XuvStitch version 1.8.099x64. Protein aggregates in each worm were detected and analyzed using Imaris x64 version 8.1.2. The surface tool was used to identify aggregates with thresholds for minimum fluorescence intensity and aggregate volume to ensure the analyzed surfaces were aggregates and not discrete proteins (**Fig S6C**): surface = 3.0, absolute intensity = 75, seed points = 5:15, intensity > 150, volume > 45, number of voxels > 500. Aggregate volume and number was exported to R for analysis.

Student's t-tests were used to determine significance in observed differences between target and EV RNAi at each age.

References

Bates D, Maechler M, Bolker B , Walker S (2015). lme4: Linear Mixed-Effects Models Using Eigen and S4ed^eds): R package version 1.1-10.

Consortium CeDM (2012). large-scale screening for targeted knockouts in the Caenorhabditis elegans genome. *G3*. **2**, 1415-1425.

Peters MJ, Joehanes R, Pilling LC, Schurmann C, Conneely KN, Powell J, Reinmaa E, Sutphin GL, Zhernakova A, Schramm K, Wilson YA, Kobes S, Tukiainen T, Consortium NU, Ramos YF, Goring HH, Fornage M, Liu Y, Gharib SA, Stranger BE, De Jager PL, Aviv A, Levy D, Murabito JM, Munson PJ, Huan T, Hofman A, Uitterlinden AG, Rivadeneira F, van Rooij J, Stolk L, Broer L, Verbiest MM, Jhamai M, Arp P, Metspalu A, Tserel L, Milani L, Samani NJ, Peterson P, Kasela S, Codd V, Peters A, Ward-Caviness CK, Herder C, Waldenberger M, Roden M, Singmann P, Zeilinger S, Illig T, Homuth G, Grabe HJ, Volzke H, Steil L, Kocher T, Murray A, Melzer D, Yaghootkar H, Bandinelli S, Moses EK, Kent JW, Curran JE, Johnson MP, Williams-Blangero S, Westra HJ, McRae AF, Smith JA, Kardia SL, Hovatta I, Perola M, Ripatti S, Salomaa V, Henders AK, Martin NG, Smith AK, Mehta D, Binder EB, Nylocks KM, Kennedy EM, Klengel T, Ding J, Suchy-Dicey AM, Enquobahrie DA, Brody J, Rotter JI, Chen YD, Houwing-Duistermaat J, Kloppenburg M, Slagboom PE, Helmer Q, den Hollander W, Bean S, Raj T, Bakhshi N, Wang QP, Oyston LJ, Psaty BM, Tracy RP, Montgomery GW, Turner ST, Blangero J, Meulenbelt I, Ressler KJ, Yang J, Franke L, Kettunen J, Visscher PM, Neely GG, Korstanje R, Hanson RL, Prokisch H, Ferrucci L, Esko T, Teumer A, van Meurs JB , Johnson AD (2015). The transcriptional landscape of age in human peripheral blood. *Nature communications*. **6**, 8570.

Satterthwaite FE (1946). An approximate distribution of estimates of variance components. *Biometrics*. **2**, 110-114.

Sutphin GL , Kaeberlein M (2009). Measuring Caenorhabditis elegans life span on solid media. *J Vis Exp*.

Sutphin GL, Mahoney JM, Sheppard K, Walton DO , Korstanje R (2016). WORMHOLE: Novel Least Diverged Ortholog Prediction through Machine Learning. *PLoS computational biology*. **12**, e1005182.
